# The Leukemia-Associated Mllt10/Af10-Dot1l Are Tcf4/β-Catenin Coactivators Essential for Intestinal Homeostasis

**DOI:** 10.1371/journal.pbio.1000539

**Published:** 2010-11-16

**Authors:** Tokameh Mahmoudi, Sylvia F. Boj, Pantelis Hatzis, Vivian S. W. Li, Nadia Taouatas, Robert G. J. Vries, Hans Teunissen, Harry Begthel, Jeroen Korving, Shabaz Mohammed, Albert J. R. Heck, Hans Clevers

**Affiliations:** 1 Hubrecht Institute and University Medical Centre Utrecht, Utrecht, The Netherlands; 2 Biomolecular Mass Spectrometry and Proteomics Group, Utrecht University, Utrecht, The Netherlands; Stanford University School of Medicine, Howard Hughes Medical Institute, United States of America

## Abstract

Wnt signaling maintains the undifferentiated state of intestinal crypt progenitor cells by inducing the formation of nuclear TCF4/β-catenin complexes. In colorectal cancer, activating mutations in Wnt pathway components cause inappropriate activation of TCF4/β-catenin-driven transcription. Despite the passage of a decade after the discovery of TCF4 and β-catenin as the molecular effectors of the Wnt signal, few transcriptional activators essential and unique to the regulation of this transcription program have been found. Using proteomics, we identified the leukemia-associated Mllt10/Af10 and the methyltransferase Dot1l as Tcf4/β-catenin interactors in mouse small intestinal crypts. Mllt10/Af10-Dot1l, essential for transcription elongation, are recruited to Wnt target genes in a β-catenin-dependent manner, resulting in H3K79 methylation over their coding regions in vivo in proliferative crypts of mouse small intestine in colorectal cancer and Wnt-inducible HEK293T cells. Depletion of MLLT10/AF10 in colorectal cancer and Wnt-inducible HEK293T cells followed by expression array analysis identifies MLLT10/AF10 and DOT1L as essential activators to a large extent dedicated to Wnt target gene regulation. In contrast, previously published β-catenin coactivators p300 and BRG1 displayed a more pleiotropic target gene expression profile controlling Wnt and other pathways. *tcf4, mllt10/af10*, and *dot1l* are co-expressed in Wnt-driven tissues in zebrafish and essential for Wnt-reporter activity. Intestinal differentiation defects in *apc*-mutant zebrafish can be rescued by depletion of Mllt10 and Dot1l, establishing these genes as activators downstream of Apc in Wnt target gene activation in vivo. Morpholino-depletion of *mllt10/af10-dot1l* in zebrafish results in defects in intestinal homeostasis and a significant reduction in the in vivo expression of direct Wnt target genes and in the number of proliferative intestinal epithelial cells. We conclude that Mllt10/Af10-Dot1l are essential, largely dedicated activators of Wnt-dependent transcription, critical for maintenance of intestinal proliferation and homeostasis. The methyltransferase DOT1L may present an attractive candidate for drug targeting in colorectal cancer.

## Abstract 2

The leukemia-associated Mllt10/Af10 and its partner the histone methyltransferase Dot1l are identified as Tcf4/β-catenin co-activators and shown to be essential for Wnt-driven endogenous gene expression, intestinal development and homeostasis.

## Author Summary

The canonical Wnt pathway is a key regulatory pathway controlling intestinal cell proliferation, differentiation, and stem cell maintenance, and its deregulation leads to malignancies in the mammalian gut. A decade has passed since the discovery of the transcription factors TCF4-β-catenin as the downstream intestinal molecular effectors of Wnt, but few transcriptional activators essential and unique to the regulation of this transcription program have been found. In this study, using a proteomics approach, we identify the leukemia-associated Mllt10/Af10 and its partner the histone methyltransferase Dot1l as interactors with Tcf4/β-catenin in the mouse small intestinal epithelium. We demonstrate that Mllt10/Af10–Dot1l are recruited to Wnt target genes in intestinal epithelial cells and are essential to regulate expression of these targets. We also show a genetic link between the Wnt pathway and Mllt10/Af10-Dot1l in zebrafish and delineate their essential role in Wnt-driven endogenous gene expression. Finally, we demonstrate the physiological role of Mllt10/Af10-Dot1l in Wnt-driven intestinal development and homeostasis; depletion of Mllt10/Af10-Dot1l in zebrafish embryos mimics the Tcf4-depleted phenotype in which significant intestinal proliferation defects accompany a decrease in total number of intestinal cells. We conclude that the enzyme Dot1l may present an attractive candidate for drug targeting in colorectal cancer.

## Introduction

The canonical Wnt signaling pathway has been shown to play a central role in cell proliferation, differentiation, and stem cell maintenance [1]. Wnt signaling controls developmental fates through the regulation of transcription of TCF/LEF target genes. β-catenin functions as a dedicated transcriptional coactivator of TCF/LEF transcription factors [2]–[4]. TCF4 constitutes the main molecular effector of this process in the intestinal epithelium [5]. In the absence of a Wnt signal, the cytosolic levels of β-catenin are kept low by a protein complex (the destruction complex) including AXIN, APC, and GSK3 [6]–[8], which induces phosphorylation of β-catenin resulting in its ubiquitination and degradation by the proteasome [9],[10]. In the absence of β-catenin, TCF4 is thought to function as a repressor of Wnt target gene expression, in part via interaction with a number of repressive cofactors such as TLE/Groucho [11],[12]. Upon Wnt signaling, the activity of the destruction complex is inhibited and β-catenin is no longer phosphorylated and translocates to the nucleus, where it interacts with TCF4 to turn on the Wnt genetic program in crypt stem/progenitor cells [5],[13].

In colorectal cancer, activating mutations in Wnt pathway components, such as APC, AXIN1, or β-catenin [14]–[16], lead to the stabilization and constitutive nuclear localization of β-catenin. The constitutive presence of TCF4/β-catenin complexes locks the Wnt transcriptional program, normally active only in crypt stem cells and progenitors, in the “on” state, resulting in malignancies in the gut.

The β-catenin/TCF4 complex is thought to drive TCF4 target gene expression by recruitment of activating cofactors [17]. Genetic approaches in *Drosophila* have led to the identification of Pygopus and Lgs/BCL9 as cofactors of β-catenin/TCF mediated transcription [18]. The c-terminus of β-catenin has been shown to bind several transcriptional coactivators, including Hyrax/Parafibromin [19]. In addition, components of the two broad classes of chromatin modulating complexes, ATP dependent chromatin remodelers and enzymes that mediate covalent modifications on histones, have been implicated in β-catenin mediated transcriptional regulation. We have previously found that the chromatin remodeling factor BRG-1 can interact with β-catenin to promote activation of certain TCF target genes [20]. Another chromatin remodeling enzyme, ISWI, and the mixed lineage leukemia H3K4 histone methyltransferase, MLL, were found to co-precipitate with the β-catenin c-terminus [21]. TBP and the acetyl transferases CBP and p300 have also been implicated in linking β-catenin to the transcription machinery, allowing access of transcription activators to target gene promoters [22]–[24]. Recently, we and others have identified the kinase TNIK as a TCF4/β-catenin interactor essential to and specific for Wnt-dependent target gene activation [25],[26].

Despite the passage of a decade after the discovery of TCF4 and β-catenin as the molecular effectors of the Wnt signal, few transcriptional activators essential and unique to the regulation of this transcription program have been found. The identification and study of the complete repertoire of nuclear TCF4/β-catenin coactivator complex components dedicated to this transcriptional program is both required to unveil the key targets and mechanistic events responsible for initiation and progression of cancer and also critical to the definition of potential therapeutic targets in colorectal cancer.

## Results

**Figure 1:**
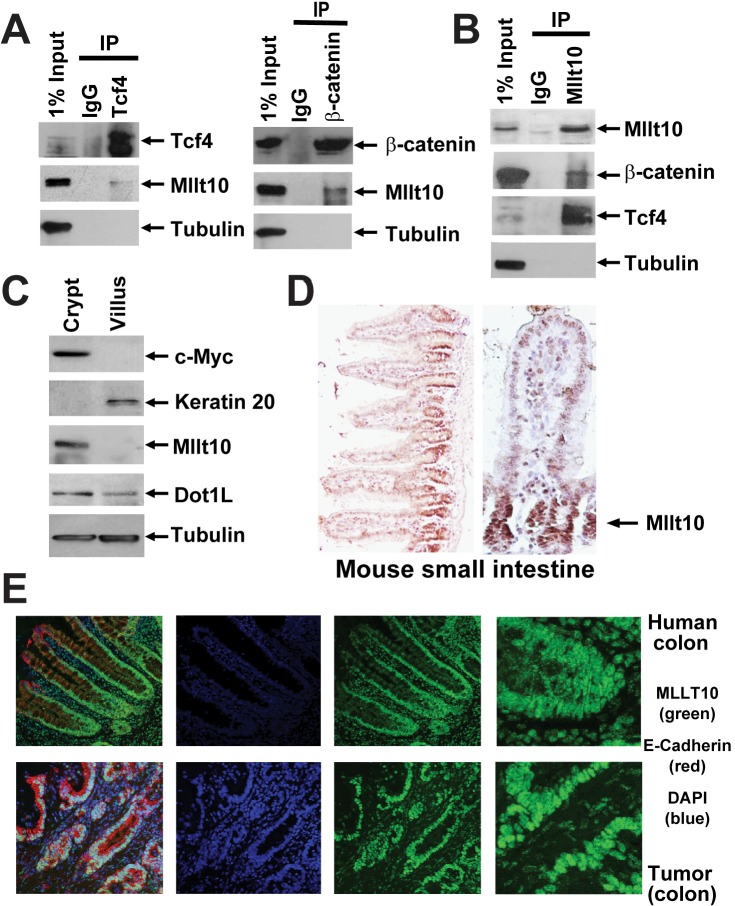
Mllt10/Af10 co-immunoprecipitates with Tcf4 in the mouse small intestinal crypt. (A) Cell lysates from purified crypt fractions were immunoprecipitated with antibodies directed against endogenous Tcf4, β-catenin, and (B) Mllt10/Af10 as indicated and analyzed by Western blotting with the indicated antibodies. (C) Western blot analysis of mechanically fractionated crypt and villus from mouse small intestine using antibodies directed against a gene expressed specifically in the crypt (c-Myc), villus (Keratin 20), Tubulin as control, as well as Mllt10/Af10 and Dot1l. (D) Mllt10/Af10 antibody staining on mouse small intestinal epithelium. Arrow indicates expression in the crypt proliferative compartment. (E) Confocal image for MLLT10/AF10 (green), E-Cadherin (red), and the nuclei counter stained with DAPi (blue). MLLT10/AF10 is expressed in nuclei of normal colon epithelium in a gradient concentrated at the crypt bottom and in colorectal cancer cells.

We applied a Mass Spectrometry approach to identify novel components of the endogenous Tcf4 complex in the mouse small intestinal epithelium. Working under the premise that intestinal crypt cells contain activating Tcf4/β-catenin complexes and that villus cells contain repressive Tcf4 complexes, we obtained separated mouse intestinal crypt and villus cells, using a mechanical fractionation method [25]. This method combines incubations of small intestine sections in chelating solutions with vigorous shaking, followed by separation of crypt and villus fractions according to size by sieving through a mesh. We confirmed the purity of the fractions by the exclusive presence of the Wnt target gene cMyc in the crypt fraction and the villus-specific marker Keratin20 in the villus compartment (Figure 1C) [25]. To identify potential Tcf4 coactivators, we immunopurified Tcf4 from purified crypt fractions and used isotype-matched IgGs as the control. The purified Tcf4 complex was subjected to SDS-PAGE and Coomassie staining, followed by Mass Spectrometry analysis of the Tcf4-associated proteins (Table S1) [25]. We identified Tcf4 and, as a positive control, β-catenin as a Tcf4 interacting protein specifically in the Tcf4 immunoprecipitations, demonstrating the validity of this approach in identifying Wnt-activated Tcf4 complexes from small intestinal crypts [25]. We thus identified the leukemia-associated Mllt10 (Af10) as an interactor of the Tcf4 complex in the crypt proliferative compartment (Figure S1A). MLLT10/AF10 was initially identified as one of the MLL translocation partners in leukemia [27]. It has also been found fused to clathrin-assembly protein-like lymphoid-myeloid (CALM) in acute myeloid leukemia (AML) and T-cell acute lymphoblastic leukemia (T-ALL) [28],[29]. MLLT10/AF10 interacts with the H3K79 histone methyltransferase DOT1L [30]. Histone modifications play an important role in the modulation of chromatin structure and gene expression. Methylation of lysine 79 of histone H3 (H3K79) is a widespread activating histone modification, which is deposited by DOT1L, the only known H3K79 methyltransferase gene in mammalian cells [31],[32]. The interaction between MLLT10/AF10 and DOT1L was proposed to underlie the main molecular mechanism of leukemogenesis by the MLL-MLLT10/AF10 and CALM-MLLT10/AF10 fusions [30],[33]; mis-targeting of DOT1L and the resulting activating H3K79 methylation causes aberrant activation of target genes, such as Hoxa5, leading to leukemia.

### Mllt10/Af10 Co-Immunoprecipitates with Tcf4 in Mouse Small Intestinal Crypts

To confirm that Mllt10/Af10 interacts with Tcf4, we immunoprecipitated Tcf4 complexes from crypt lysates and probed for the association with endogenous Mllt10/Af10 by Western blotting using an antibody specific for Mllt10/Af10. The Tcf4 complex contained Mllt10/Af10 in the crypts of the small intestinal epithelium (Figure 1A left panel). Immunoprecipitation of β-catenin complexes from crypts followed by Western blot analysis confirmed the association with Mllt10/Af10 (Figure 1A right panel). The same interactions were also detected and confirmed in reverse Western blot analysis of the Mllt10/Af10-associated proteins immunoprecipitated from crypts using antisera directed against Mllt10/Af10 (Figure 1B). These results demonstrated that Mllt10/Af10 specifically interacts with Tcf4/β-catenin complexes in mouse small intestinal crypts.

### Mllt10/Af10 Is Expressed in Crypt (But Not Villus) Compartments

In the mouse small intestine, Mllt10/Af10 was found to be expressed specifically in the crypt proliferative compartment and not the villus as shown by Western blotting on purified crypt and villus fractions (Figure 1C) and by immunohistochemistry (Figure 1D). In healthy human colon, MLLT10/AF10 was expressed in a decreasing gradient with the highest concentration at the bottom of the crypts (Figure 1E top panel). MLLT10/AF10 was also highly expressed in human colon tumor samples (Figure 1E bottom panel). The expression of MLLT10/AF10 specifically in the proliferative crypt compartment was consistent with its potential role as an activator of Wnt dependent transcription.

### Specific Enrichment of Mllt10/Af10, Dot1l, and H3K79 Methylation at Wnt Target Genes in Proliferative Crypts of Mouse Small Intestine

**Figure 2:**
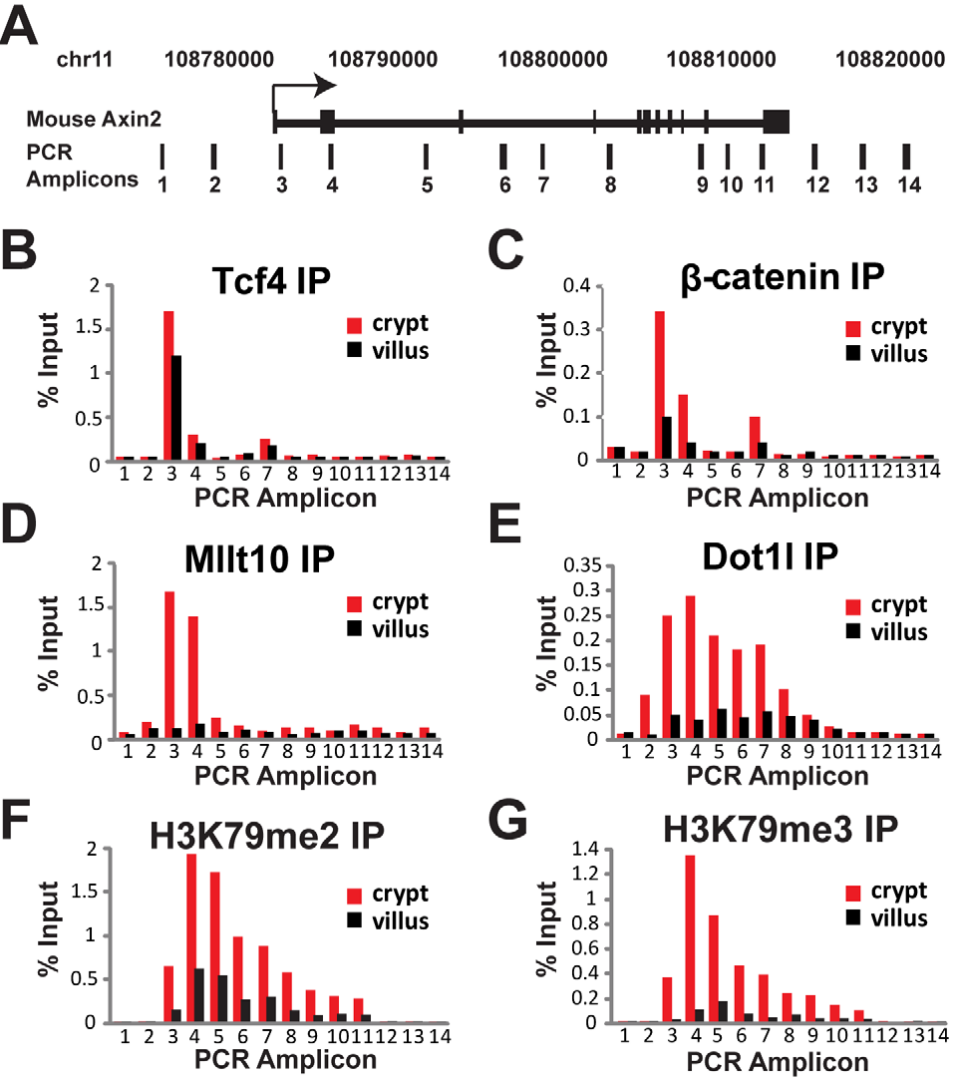
Specific enrichment of Mllt10/Af10 and Dot1l, and H3K79 methylation at *Axin2* regulatory regions in mouse proliferative crypts. (A) Schematic representation of the mouse *Axin2* locus and amplicons scanned in Chromatin immunoprecipitation experiments by qPCR. Purified crypt and villus fractions from mouse small intestine were subjected to ChIP using antibodies against Tcf4 (B), β-catenin (C), Mllt10/Af10 (D), Dot1l (E), H3K79 dimethyl (F), and H3K79 trimethyl (G). Chromatin was immunoprecipitated with the specified antibodies followed by qPCR using primer pairs spanning the mouse *Axin2* locus as indicated in (A). Results are presented as percentage immunoprecipitated over input and are representative of three independent experiments.

To test whether Mllt10/Af10 interacts with Tcf/β-catenin target gene chromatin in vivo, we performed chromatin immunoprecipitation (ChIP) assays on mouse small intestinal epithelium (Figure 2 and Figure S2). The purified crypt and villus fractions consist of single-cell layers, allowing direct formaldehyde fixation, an essential step in ChIP. We performed direct conventional ChIP coupled to quantitative PCR (qPCR) and confirmed the technical effectiveness of ChIP on isolated crypts and villi. We designed primers spanning the upstream regulatory regions, gene body, and downstream regulatory regions of the well-described Wnt target genes mouse *Axin2* (Figure 2A) and mouse *c-Myc* (Figure S2A). Chromatin prepared from crypt and villus was subjected to ChIP with antibodies specific for Tcf4, β-catenin, and Mllt10/Af10. As the histone H3K79 methyltransferase, and Mllt10/Af10 interacting partner, Dot1l was also identified in the crypt Tcf4 complex by Mass Spectrometry, although with a less robust score (Figure S1B), we also performed ChIPs with antibodies specific for Dot1l, as well as for the activating H3K79 di- and tri-methyl marks.

qPCR analysis of the immunoprecipitated material with primers specific for the mouse *Axin2* and *c-Myc* loci indicated that Mllt10/Af10 and Dot1l were specifically recruited to the promoters of Wnt target genes *Axin2* and *c-Myc* in the proliferative crypt and not the differentiated villus compartment (Figure 2D and 2E and Figure S2D and S2E). Mllt10/Af10 enrichment was specific to the Tcf4-occupied regions of *Axin2* and *c-Myc* and not the non-bound upstream and downstream control regions. Dot1l enrichment was also observed at the Tcf4-bound regions and additionally spanned the transcribed gene body (Figure 2E and Figure S2E). As expected, Tcf4 was bound specifically to the target genes in both the crypt and villus while β-catenin was enriched on the target promoters specifically in the crypts (Figure 2B and 2C and Figure S2B and S2C). Importantly, the activating H3K79 di- and tri-methyl marks were enriched on the Wnt target gene transcribed regions specifically in the crypt, mimicking Dot1l enrichment (Figure 2F and 2G and Figure S2F and S2G). Together, these results agreed with the notion that Mllt10/Af10 and Dot1l are part of the Tcf4/β-catenin transcriptional complex in vivo.

### Recruitment of MLLT10/AF10-DOT1L to Wnt Target Genes Is β-catenin Dependent

**Figure 3:**
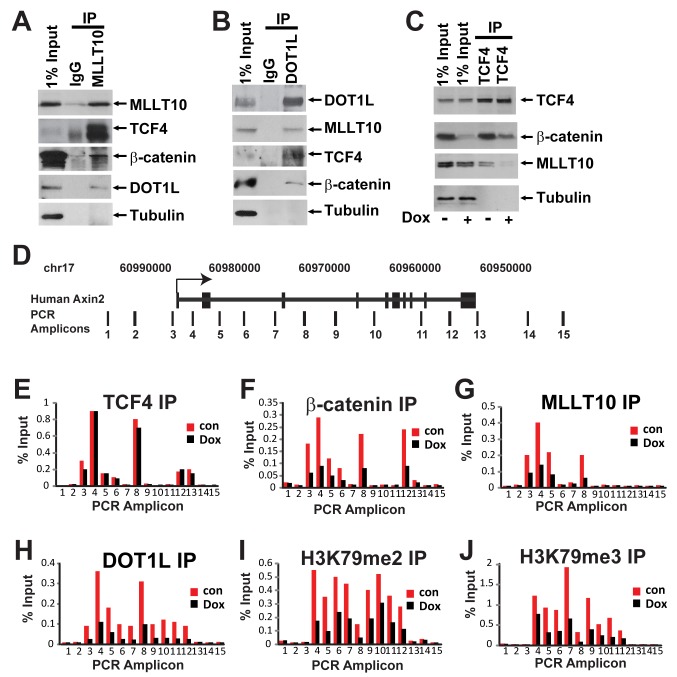
β-catenin-dependent H3K79 methylation and MLLT10/AF10, DOT1L recruitment to human *AXIN2* gene. Cell lysates from Ls174T CRCs were immunoprecipitated with antibodies against endogenous MLLT10/AF10 (A) and DOT1L (B) complexes and analyzed by Western blotting with the indicated antibodies. (C) MLLT10/AF10 interaction with TCF4 is mediated by β-catenin. Western blot analysis of β-catenin depletion in Ls174T cells expressing doxycycline (Dox)-inducible β-catenin shRNA. Immunoprecipitated TCF4-protein complexes from untreated or Dox-treated cells were resolved by SDS-PAGE followed by Western blotting with the indicated antibodies. (D) Schematic representation of the human *AXIN2* locus and amplicons scanned in Chromatin immunoprecipitation experiments by qPCR. ChIP in Ls174T CRCs uninduced or induced with Dox using antibodies specific for TCF4 (E), β-catenin (F), MLLT10/AF10 (G), DOT1L (H), H3K79 dimethyl (I), and H3K79 trimethyl (J). The immunoprecipitated DNA was analyzed by qPCR using primers specific for the *AXIN2* locus as indicated. Results are presented as percent immunoprecipitated over input and are representative of three independent experiments.

We next examined the interaction between TCF4, β-catenin, and MLLT10/AF10-DOT1L in the Ls174T colorectal carcinoma cell line (CRC). In this cell line a point mutation in β-catenin results in its constitutive stabilization and activation of the Wnt pathway. This Wnt “on” system mimics the proliferative crypt compartment, in which the Wnt pathway is active. MLLT10/AF10 was immunoprecipitated from Ls174T CRCs and probed for interaction with TCF4, β-catenin, and DOT1L by Western blotting (Figure 3A). Both TCF4 and β-catenin specifically associated with MLLT10/AF10, highlighting the conserved nature of this interaction. Similarly, DOT1L was immunoprecipitated from Ls174T cells and probed for interaction with TCF4, β-catenin, and MLLT10/AF10 by Western blotting. Again, TCF4 and β-catenin were present in the DOT1L complex, and as expected, MLLT10/AF10 also interacted with DOT1L (Figure 3B). Conversely, MLLT10/AF10 and DOT1L were detected in immunoprecipitates of TCF4 and β-catenin from Ls174T CRCs, confirming the reciprocal interaction (Figures S3A).

To examine whether the TCF4-MLLT10/AF10-DOT1L interaction is mediated by β-catenin, we used an Ls174T CRC cell line that inducibly expresses a shRNA against β-catenin in response to doxycycline treatment [5]. In these cells, β-catenin is significantly depleted at 72 h post-doxycycline treatment (Figure 3C). This depletion results in abrogation of the constitutive transcriptional activity of the Wnt pathway. We immunoprecipitated TCF4 from Ls174T cells untreated or treated with doxycycline and probed for the presence of MLLT10/AF10 in the transcriptional complex. Depletion of β-catenin resulted in loss of binding of MLLT10/AF10 to TCF4, suggesting that β-catenin bridges the interaction between TCF4 and MLLT10/AF10 (Figure 3C). Using the same system we then examined the recruitment of TCF4, β-catenin, MLLT10/AF10, DOT1L, and H3K79 methylation on the human TCF4 target genes *AXIN2* and *c-MYC* in vivo by ChIP in the presence or absence of β-catenin (Figure 3D–3J and Figure S3B–S3H). We designed primers spanning the upstream region, gene body, and downstream region of human *AXIN2* and *c-MYC* genes (Figure 3D and Figure S3B). As observed previously in the mouse small intestinal epithelium, TCF4 is present on the *AXIN2* and *c-MYC* regulatory regions in Ls174T CRCs [34] and not the control upstream/downstream unbound regions regardless of the presence or absence of β-catenin (Figure 3E and Figure S3C). As expected, removal of β-catenin by shRNA resulted in its removal from the *AXIN2* and *c-MYC* loci (Figure 3F and Figure S3D). Importantly, while MLLT10/AF10-enrichment mimicked TCF4 binding on the *AXIN2* and *c-MYC* loci in the presence of β-catenin, this enrichment was abrogated upon β-catenin depletion (Figure 3G and Figure S3E). Similarly to its binding pattern in the mouse small intestinal crypts, DOT1L recruitment to *AXIN2* and *c-MYC* target genes was enriched on TCF4 bound regions and spanned the transcribed gene body. Again, DOT1L enrichment was β-catenin dependent (Figure 3H and Figure S3F). The pattern of enrichment of the activating H3K79 di- and tri-methyl marks mimicked DOT1L binding; depletion of β-catenin reduced di-/tri-methylation of H3K79 on *AXIN2* and *c-MYC* (Figure 3I and 3J and Figure S3G and S3H). Therefore, we concluded that MLLT10/AF10-DOT1L recruitment to the Wnt target genes *AXIN2* and *c-MYC* is mediated by β-catenin. To determine whether MLLT10/AF10 interacts directly with β-catenin, we generated recombinant GST fusion constructs of TCF4, β-catenin, as well as C-terminal and N-terminal MLLT10/AF10. We performed GST-pulldown assays with in vitro transcribed and translated S^35^ labeled MLLT10/AF10 and β-catenin deletion mutants (Figure S4A–S4C). We found that MLLT10/AF10 directly interacts with the β-catenin C-terminal domain.

### MLLT10/AF10 Is an Essential Activator Dedicated to TCF-Driven Activation of Wnt Targets

**Figure 4:**
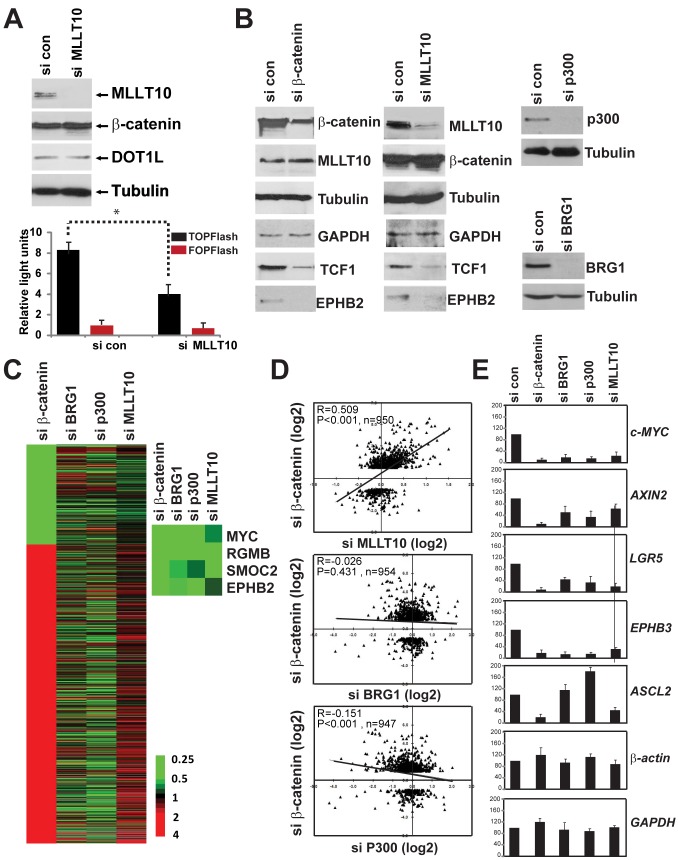
MLLT10/AF10 is an essential activator dedicated to TCF-driven activation of endogenous Wnt target genes. (A) Loss of MLLT10/AF10 abrogates TCF/LEF driven transcriptional activation. siRNA mediated depletion of MLLT10/AF10 reduces β-catenin/TCF driven transcription in Ls174T CRCs. Activity of the integrated TOPFlash (black bars) and FOPFlash (red bars) is shown. Error bars represent standard deviation from three independent experiments (*) *p* value = 0.009. Expression of MLLT10/AF10, β-catenin, DOT1L, and control Tubulin was analyzed by Western blotting after depletion of MLLT10/AF10. (B) Western blot analysis of MLLT10/AF10, β-catenin, BRG1, p300, and the endogenous TCF4 targets TCF1 and EPHB2, and control Tubulin and GAPDH protein levels in Ls174T CRCs after siRNA depletion of MLLT10/AF10 or β-catenin as indicated. (C) Heat map showing 1003 Wnt regulated transcripts (more than 2-fold variation upon *β-catenin* depletion) in Ls174T CRCs, and comparison of corresponding expression pattern after siRNA suppression of *BRG1*, *p300*, and *MLLT10/AF10*. Green, down-regulated after *β-catenin* suppression; red, upregulated after *β-catenin* suppression; grey, missing data. Representatives of known Wnt target genes are listed on the right. (D) Pearson correlation of Wnt target genes upon *β-catenin* knockdown (*x*-axis: fold change (log2)) and after siRNA suppression of *MLLT10/AF10* (top), *BRG1* (middle), or *p300* (bottom) (*y*-axis: fold change (log2)). (E) Relative expression levels of Wnt target genes *c-MYC*, *AXIN2*, *LGR5*, *EPHB3*, and *ASCL2* in Ls174T CRC by RT-qPCR of RNA isolated 85 h after siRNA transfection. Bar graphs represent the mean of two independent experiments, each analyzed in triplicate by RT-qPCR.

The expression of Mllt10/Af10 specifically in the proliferative crypt intestinal compartment and its recruitment to Wnt target genes in a β-catenin-dependent manner were consistent with its function as a β-catenin-recruited coactivator. To test this, we depleted MLLT10/AF10 from Ls174T CRCs containing an integrated Tcf reporter using siRNA specifically targeting MLLT10/AF10 (Figure 4A). Significant depletion of MLLT10/AF10 was achieved while β-catenin and DOT1L expression was unaffected as shown by Western blotting (Figure 4A top panel). Removal of MLLT10/AF10 by siRNA resulted in specific suppression of the integrated TCF luciferase reporter (“TOP”) activity, while activity of the mutant renilla reporter (“FOP”) remained unaffected (Figure 4A bottom panel). Thus, MLLT10/AF10 is required for optimal TCF/LEF driven transcription.

We next examined the expression levels of several TCF4/β-catenin endogenous target genes upon depletion of MLLT10/AF10 or β-catenin, the latter as a positive control, from Ls174T cells (Figure 4B). Western blot analysis indicated that β-catenin and MLLT10/AF10 were efficiently depleted by the respective siRNAs. As expected, depletion of β-catenin resulted in down-regulation of the TCF4 target genes TCF1 [35] and EPHB2 [36] (Figure 4B left panel). Consistent with its role as an activator of Wnt target genes, depletion of MLLT10/AF10 also resulted in down-regulation of the endogenous TCF4 target genes TCF1 and EPHB2, while the controls Tubulin and GAPDH were not affected (Figure 4B right panel).

To examine the specificity of MLLT10/AF10 in Wnt transcriptional regulation, we performed microarray analysis in Ls174T colorectal cancer cells comparing the effect of MLLT10/AF10 depletion with two other known Wnt coactivators, BRG1 [20] and p300 [22]. The Wnt target gene program in Ls174T CRCs was identified by the resulting differential gene expression pattern (genes with greater than 2-fold variation) following depletion of β-catenin by siRNA. With this criterion 1,003 transcripts were identified representing 827 unique genes (Figure 4C and Table S2). Comparison of the corresponding expression pattern of these Wnt target genes in the absence of MLLT10/AF10, BRG1, and p300, respectively, shows that β-catenin suppression shares better expression pattern similarity and correlation with MLLT10/AF10 depletion (Pearson correlation coefficient = 0.509) than BRG1 (Pearson correlation coefficient = −0.026) or p300 (Pearson correlation coefficient = −0.151) (Figure 4D). Despite the poor correlation of BRG1 and p300 with Wnt regulation (si β-catenin), several known Wnt target genes such as c-*MYC*, *RGMB*, *SMOC2*, and *EPHB2* were found to be downregulated in all four panels (Figure 4C). Our data suggest that while all three transcription coactivators are required for activation of certain Wnt target genes, MLLT10/AF10 demonstrates a higher specificity for Wnt transcription regulation than BRG1 and p300. To validate the microarray data in Ls174T CRC, we chose five representative genes—*c-MYC*, *AXIN2*, *LGR5*, *EPHB3*, and *ASCL2*—for quantitative RT-PCR validation (Figure 4E). All selected genes were downregulated upon depletion of MLLT10/AF10 and as a positive control β-catenin, while most but not all (*ASCL2*) Wnt targets were downregulated upon BRG1 and p300 depletion.

### MLLT10/AF10 and DOT1L Are Essential for and Largely Dedicated to the Wnt-Induced Transcriptional Program in the HEK293T System

**Figure 5:**
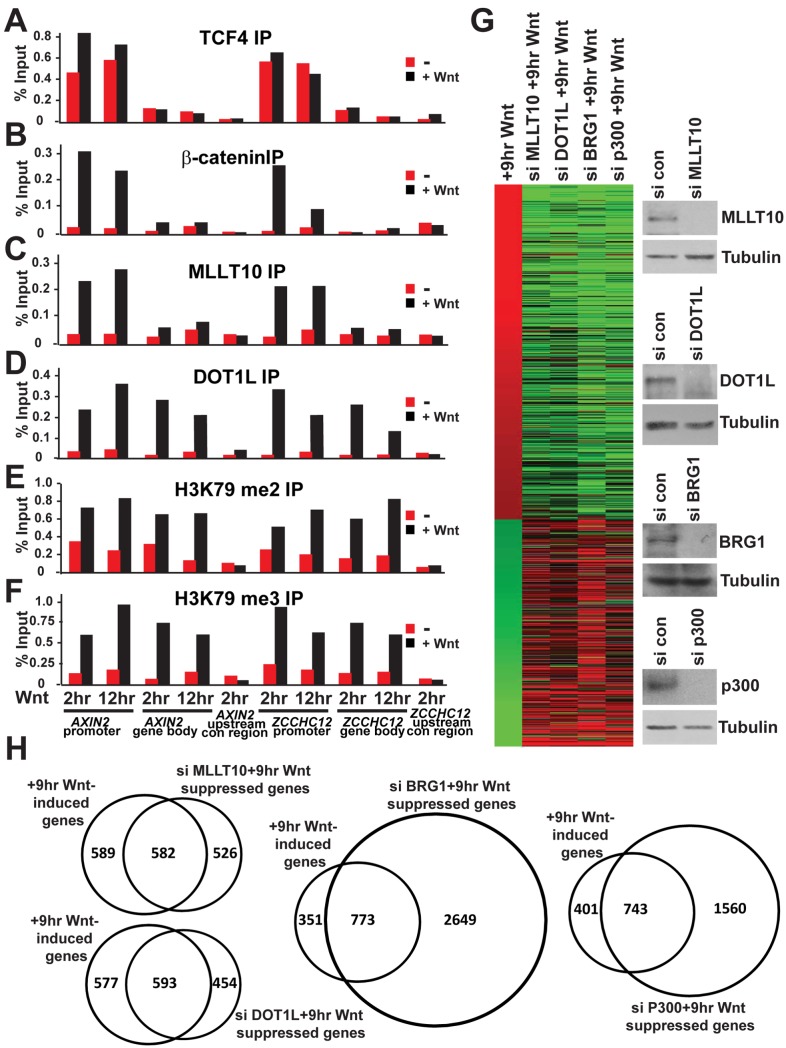
MLLT10/AF10 and DOT1L are essential and dedicated to the Wnt-induced transcriptional program in HEK293T cells. Wnt-induced association of MLLT10/AF10-DOT1L with and regulation of Wnt target genes in HEK293T cells. (A–F) ChIP assays in HEK293T cells uninduced or induced with Wnt3A conditioned media at 2 and 12 h using antibodies specific for TCF4 (A), β-catenin (B), MLLT10/AF10 (C), DOT1L (D), H3K79 di-methyl (E), and H3K79 tri-methyl (F). The immunoprecipitated DNA was analyzed by qPCR using primers specific for the *c-MYC* and *ZCCHC12* loci as indicated. Results are presented as percent immunoprecipitated over input and are representative of three independent experiments. (G) Comparison of the corresponding expression pattern after siRNA suppression of MLLT10/AF10, DOT1L, BRG1, and p300 in the Wnt induced condition. Heatmap showing 1988 Wnt regulated transcripts after 9 h Wnt-induction (relative to uninduced sample (no Wnt)) in HEK293T cells with greater than 1.5-fold variation, and the comparison of the corresponding expression pattern after siRNA suppression of MLLT10/AF10, DOT1L, BRG1, and p300 in Wnt-induced condition (relative to 9 h Wnt induction). Red, upregulated after Wnt; green, downregulated after Wnt induction; grey, missing data. Western blot analysis of MLLT10/AF10, DOT1L, BRG1, p300, and Tubulin upon siRNA depletion of each gene as indicated. (H) Venn diagram depicting the comparison of Wnt-induced genes and genes downregulated after MLLT10/AF10, DOT1L, BRG1, or p300 suppression in HEK293T cells after Wnt induction.

Our data using the constitutive Wnt activated Ls174T CRC demonstrated that depletion of MLLT10/AF10-DOT1L reduced Wnt target gene expression. To demonstrate that activation of Wnt signaling in unstimulated cells results in a time-dependent association of MLLT10/AF10 and DOT1L on Wnt target gene promoters, we extended our studies to the physiologically relevant HEK293T system. In the latter, Wnt pathway components are intact but not mutationally activated. Wnt stimulation in these cells results in a time-dependent activation of a Wnt target gene program. ChIP analysis in HEK293T cells indicates that while TCF4 is enriched on the Wnt target genes *AXIN2* and *ZCCHC12* [25], in the absence or presence of Wnt, recruitment of β-catenin, MLLT10/AF10, DOT1L, and H3K79 di-/tri-methylation occurs in a Wnt dependent manner (Figure 5A–5F). We performed siRNA-depletion of BRG1, p300, and MLLT10/AF10 in HEK293T cells stimulated with Wnt followed by microarray analysis. Unlike Ls174T cells in which depletion of DOT1L resulted in cell death, HEK293T cells appeared healthy upon DOT1L depletion. Therefore, we were also able to examine the requirement and specificity of DOT1L in Wnt target gene regulation in this system. Western blot analysis indicated efficient depletion of each protein in response to its corresponding siRNA (Figure 5G right panel). Transcripts representing 1,714 unique genes were identified as Wnt targets after stimulation with Wnt (Figure 5G and 5H and Table S3). Comparison of the corresponding Wnt signature following depletion of MLLT10/AF10, DOT1L, BRG1, and p300 showed that Wnt induced genes were generally down-regulated in the absence of each coactivator. However, comparison of the overlapping percentage of Wnt-induced genes with those suppressed upon depletion of MLLT10/AF10, DOT1L, BRG1, or p300 demonstrated that over half of MLLT10/AF10 and DOT1L-suppressed genes (53% and 57%, respectively) are Wnt targets, while only 20% and 30%, respectively, of BRG1 and p300 suppressed genes are Wnt-induced (Figure 5H). Thus, while the chromatin modifiers BRG1 and p300 are functionally pleiotropic and largely involved in other gene regulatory pathways, MLLT10/AF10-DOT1L show greater specificity for regulation of Wnt targets. As expected, comparison of the corresponding gene expression pattern after siRNA depletion of MLLT10/AF10 and DOT1L in HEK293T cells under Wnt stimulation indicates that they share significant expression pattern similarity and over 60% of their target genes (Figure S5A and S5B).

### MLLT10/AF10 Is Essential for Transcription Elongation Linking DOT1L and H3K79 Methylation to TCF4/β-Catenin Target Gene Chromatin

**Figure 6:**
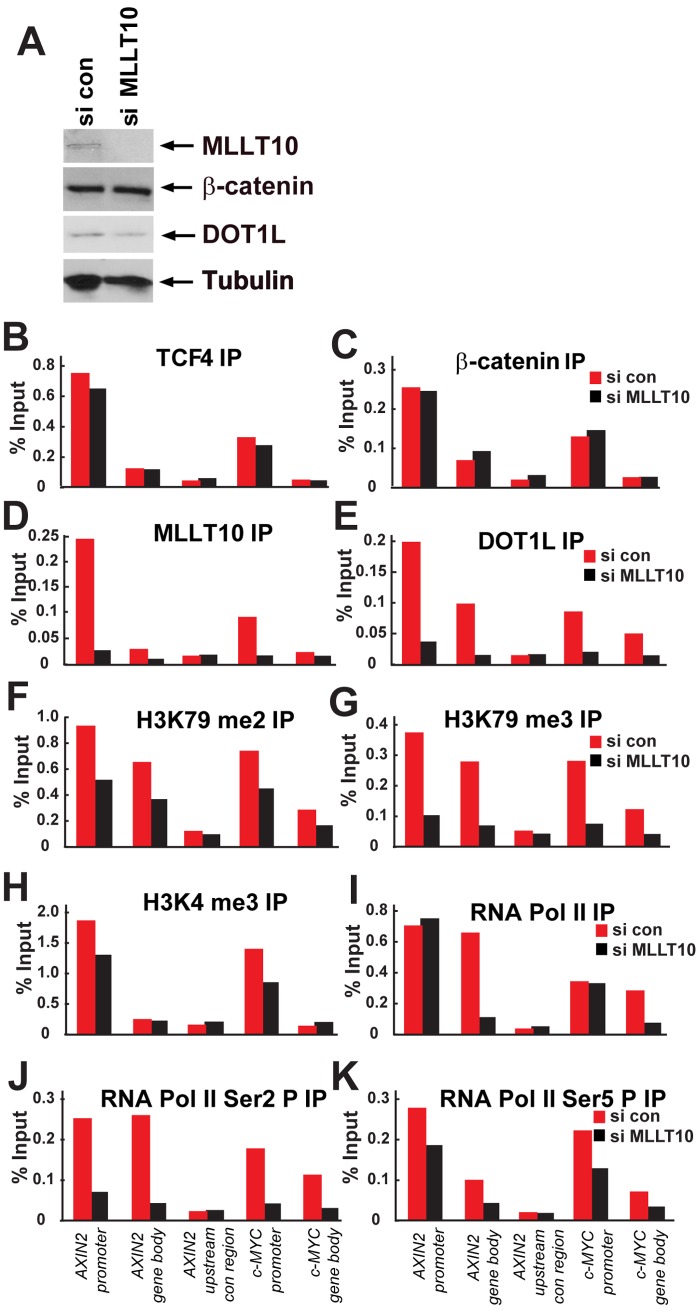
MLLT10/AF10 targets DOT1L-H3K79 methylation to Wnt target genes and is essential for transcription elongation. (A) Expression levels of MLLT10/AF10, β-catenin, DOT1L, and Tubulin analyzed by Western blotting after siRNA depletion of MLLT10/AF10. ChIP experiments in Ls174T CRCs containing or depleted of MLLT10/AF10 by siRNA using antibodies against (B) TCF4, (C) β-catenin, (D) MLLT10/AF10, (E) DOT1L, (F) H3K79 dimethyl, (G) H3K79 trimethyl, (H) H3K4trimethyl, (I) RNA Pol II, (J) RNA Pol II Ser2P, and (K) RNA Pol II Ser5P. Immunoprecipitated DNA was analyzed by qPCR using primers specific for *AXIN2* and *c-MYC* promoters and unbound *AXIN2* upstream control region. Results are presented as percent immunoprecipitated over input and are representative of three independent experiments.

To address whether the recruitment of DOT1L and the activating H3K79 di-/tri-methylation mark to TCF4/β-catenin target genes is mediated by MLLT10/AF10, we depleted MLLT10/AF10 from Ls174T CRC and performed ChIP probing recruitment of DOT1L and H3K79 di-/tri-methylation on *AXIN2* and *c-MYC* (Figure 6). Western blot analysis shows significant siRNA depletion of MLLT10/AF10 while β-catenin and DOT1L levels were unaffected (Figure 6A). As expected, enrichment of TCF4 and β-catenin to the Wnt targets was unaffected upon MLLT10/AF10 depletion (Figure 6B,C). However, upon depletion of MLLT10/AF10, DOT1L recruitment and consequent H3K79 di-/tri-methylation were decreased, indicating that MLLT10/AF10 bridges DOT1L to TCF4/β-catenin target genes (Figure 6D–G).

To dissect the mechanism by which MLLT10/AF10 transcriptionally activates Wnt target genes, we examined the association of the elongating RNA pol II [37] and H3K4 trimethylation, a mark of actively transcribed genes [38] on Wnt target genes (Figure 6H–K). We found that while the promoters are primed with RNA Pol II and Ser5 phosphorylated RNA Pol II in the presence or absence of MLLT10/AF10 (Figure 6I,K), recruitment of elongating Ser2 phosphorylated RNA Pol II to *AXIN2* and *c-MYC* was abolished upon MLLT10/AF10 depletion (Figure 5J). Depletion of MLLT10/AF10 did not significantly affect the enrichment of the activating H3K4 trimethyl mark on the Wnt target promoters (Figure 5H). These results demonstrate that MLLT10/AF10 is essential for transcriptional elongation, and not initiation of transcription at Wnt target genes.

### *tcf7l2/tcf4*, *mllt10/af10*, and *dot1l* Are Expressed in Wnt Active Tissues in Zebrafish

Our data indicated a critical role for MLLT10/AF10-DOT1L as activators of TCF4/β-catenin target genes. To demonstrate the function of MLLT10/AF10-DOT1L in Wnt signaling and intestinal development in vivo, we used zebrafish (*Danio rerio*) as a model system. Zebrafish has been shown to be an important and relevant model system for the study of development of the intestinal epithelium and tumorigenesis [39]–[41]. *tcf4/tcf7l2* mutant zebrafish were shown to recapitulate the phenotype of the *Tcf4* knockout mouse [42], i.e. the loss of proliferation in the intestine [40]. Thus, *tcf4/tcf7l2* plays a similar critical role in zebrafish intestinal development as it does in mouse. Using the Mllt10/Af10 mouse protein sequence, we identified the full zebrafish Mllt10/Af10 protein sequence (Figure S6A). Inspection of the protein alignments indicated that the main functional domains of Mllt10/Af10, the PHD fingers, and the LZ domain, which mediates binding to Dot1l [33], are highly conserved across species (Figures S6B and S6C). The zebrafish Mllt10/Af10 Leucine-zipper and PHD finger domains were 74.5% and 72.4% identical to human and mouse sequences, respectively.

**Figure 7:**
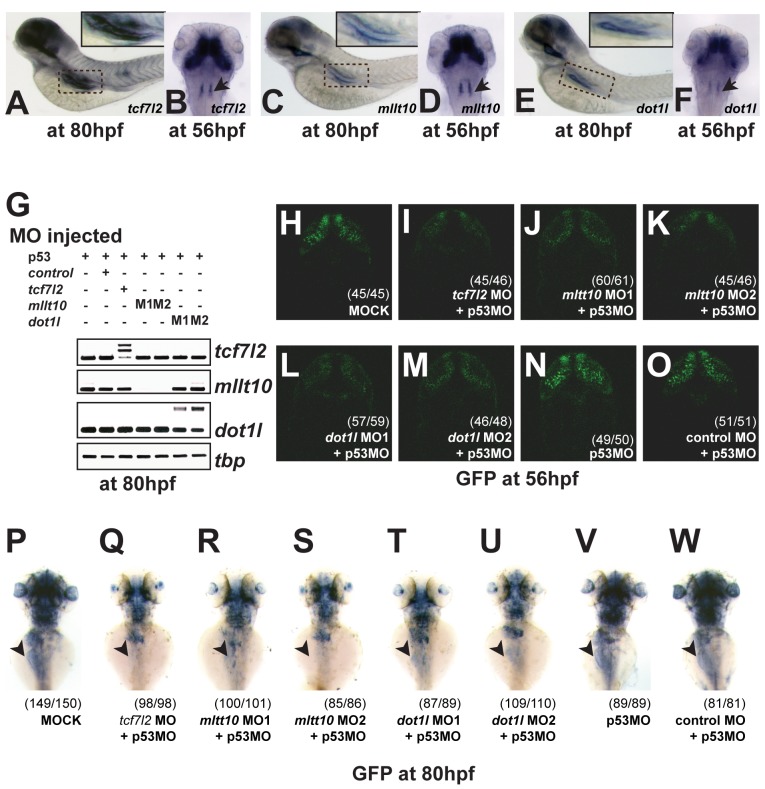
*tcf7l2*, *Mllt10/Af10*, and *dot1l* are co-expressed in Wnt active tissues and required for TCF-driven transcription in vivo. (A–F) In situ hybridization for *tcf7l2* (A), *mllt10/af10* (C), and *dot1l* (E) in whole embryos at 80 hpf. In situ hybridization for *tcf7l2* (B), *mllt10/af10* (D), and *dot1l* (F) in whole embryos at 56 hpf. Arrowhead indicates rhombomeres. (G) Gene-specific depletion of RNA levels at 80 hpf determined by RT-PCR analysis of *tcf7l2, mllt10*, and *dot1l* expression in whole embryos injected with the indicated MO sequences. Higher bands in the RTPCR panels for *tcf7l2* and *dot1l* correspond to unspliced introns in the PCR products. Morpholino knockdown of *mllt10/af10* and *dot1l* mimics *tcf7l2* depletion effect on Wnt reporter activity in zebrafish brain (H–M). Representative confocal images of dorsal view of head of 56 hpf TOPdGFP embryos injected with different MOs. (H) dGFP expression in the hindbrain of embryos injected with buffer only, MO against *tcf7l2* (I), two independent *mllt10/af10* (J,K) and *dot1l* MOs (L,M), *p53* MO (N), and control MO (O). Depletion of *mllt10/af10* and *dot1l* abrogate GFP expression in intestine of TOPdGFP zebrafish, mimicking *tcf7l2* depletion. Representative dorsal view of whole mount in situ hybridization for *GFP* in heterozygous TOPdGFP embryos at 80 hpf injected with (P) buffer alone, MOs against *tcf7l2* (Q), *mllt10/af10* (R,S), *dot1l* (T,U), *p53* (V) and control MO (W). All MOs have been coinjected with a MO against *p53*.

RT-PCR analysis of *tcf7l2*, *mllt10/af10*, and *dot1l* using RNA harvested from zebrafish embryos at various time points between 0 and 80 h post-fertilization (hpf) demonstrated expression of *tcf7l2* in embryos by 13 hpf, which remained strong at all subsequent time points examined (Figure S6D). *mllt10/af10* was expressed later than *tcf7l2*, at 24 hpf, while *dot1l* expression was already detected at 2 hpf (Figure S6D). We determined the expression pattern of *tcf7l2*, *mllt10/af10*, and *dot1l* in zebrafish at 56 and 80 hpf, developmental time points at which all genes are clearly expressed (Figure 7A–7F). In situ hybridization analysis revealed that *tcf7l2* is clearly expressed in the intestine at 80 hpf (Figure 7A) and at 56 hpf in the midbrain, and three rhombomeres in the hindbrain as seen before [43], areas of known Wnt activity (Figure 7B). Strikingly, in situ hybridization for *mllt10/af10* and *dot1l* revealed the same expression pattern in Wnt-driven tissues, in the intestine at 80 hpf and brain at 56 hpf (Figure 7C–7F).

### *mllt10/af10* and *dot1l* Are Required for the Tcf-Driven Wnt Transcriptional Program In Vivo

The TOPdGFP transgenic line, a faithful reporter line of Wnt signaling, expresses GFP under the control of TCF/LEF binding sites in regions of active Wnt signaling [44]. In these embryos, GFP was highly expressed in the brain (Figure 7H, 7N, and 7O) and moderately in the intestine (Figure 7P, 7V, and 7W). To investigate the role of *tcf7l2*, *mllt10/af10*, and *dot1l* in Wnt/TCF-driven transcription in vivo, we designed morpholinos (MO) to deplete these genes (Figure S6E). RT-PCR analysis confirmed the efficiency and specificity of the different MOs used to deplete *tcf7l2*, *mllt10/af10* (two independent sequences), and *dot1l* (two independent sequences) (Figure 7G). In all experiments, we co-injected a p53 MO [45] in order to control for potential off-target effects arising from MO injection. *tcf7l2* depletion caused a striking decrease in GFP expression in the midbrain of heterozygous TOPdGFP embryos at 56 hpf (Figure 7I), indicating that this Tcf member is required for the expression of the GFP reporter in vivo, as has already been reported for *lef1* [44], while injection of a control unrelated MO had no effect on midbrain GFP expression (Figure 7O). Similarly, depletion of *mllt10/af10* and *dot1l* resulted in a decrease in GFP expression in the midbrain (Figure 7J–7M), highlighting their requirement for TCF-driven transcription in vivo.

The GFP fluorescence signal in the intestine of TOPdGFP fish was too weak to be detected directly by microscopy. Therefore, we analyzed the effect of *tcf7l2*, *mllt10/af10*, and *dot1l* depletion on Wnt activity in the intestine of TOPdGFP embryos by in situ hybridization for GFP (Figure 7P–7W). While injection of a control MO displayed robust intestinal GFP expression (Figure 7V and 7W), MO depletion of *tcf7l2* (Figure 7Q), *mllt10/af10* (Figure 7R and 7S), and *dot1l* (Figure 7T and 7U) resulted in loss of intestinal GFP expression. These results confirmed Mllt10/Af10-Dot1l as essential members of the Tcf-driven transcriptional complex in Wnt-active tissues in zebrafish.

Control morpholino injected TOPdGFP embryos showed strong expression of GFP within the hindbrain region and the lens, as seen previously [44]. Consistent with the presence of other *tcf/lef* members in the brain [44],[46], knockdown of *tcf7l2* did not completely abolish the expression of GFP in the hindbrain (Figure 7I). This same effect was observed after depletion of *mllt10/af10* and *dot1l* by morpholino injection (Figure 7J–7M).

### Depletion of *mllt10* and *dot1l* Rescues Intestinal Defects in *apc min* Zebrafish, Mimicking *tcf7l2* Depletion

**Figure 8:**
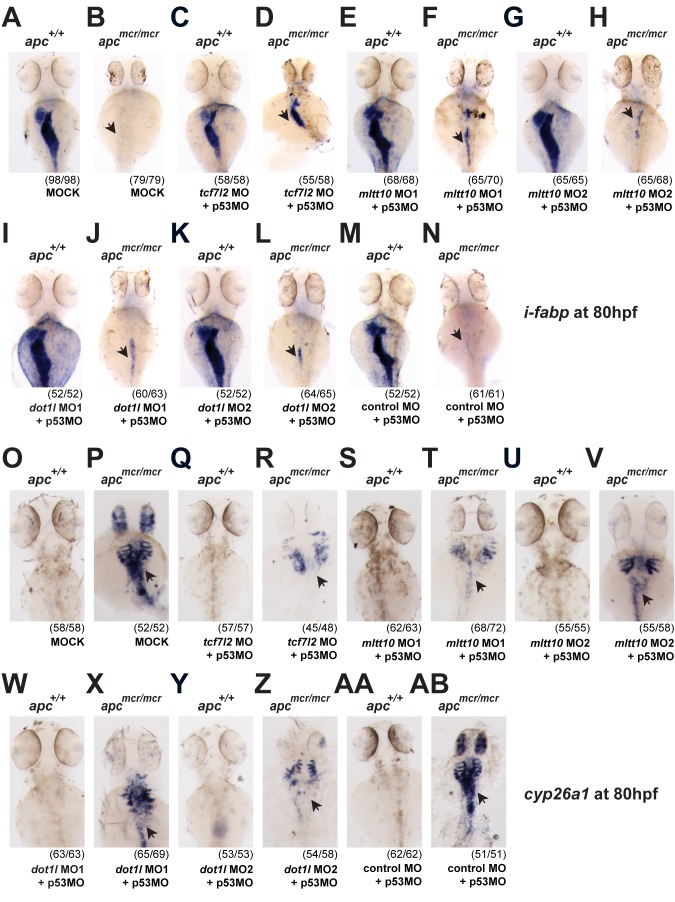
Depletion of *mllt10/af10* and *dot1l* rescues intestinal defects in apc^mcr/mcr^ zebrafish, mimicking tcf7l2 depletion. Depletion of *mllt10/af10* and *dot1l* rescues expression of *i-fabp* in *apc^mcr/mcr^* mutant embryos, placing these genes downstream of Apc as Wnt target gene activators (A–N). Representative whole mount in situ hybridizations for *i-fabp* in wild type and *apc^mcr/mcr^* mutant embryos at 80 hpf injected with (A,B) buffer alone,(C,D) MO against *tcf7l2*, (E–H) two independent *mllt10/af10* MOs, (I–L) two independent *dot1l* MO, and (M,N) control MO. Depletion of *mllt10/af10* and *dot1l* rescues mis-expression of *cyp26a1* in *apc^mcr/mcr^* mutant embryos (O–AB). Representative whole mount in situ hybridizations for *cyp26a1* in wild type and *apc^mcr/mcr^* mutant embryos at 80 hpf injected with (O,P) buffer alone, (Q,R) MO against *tcf7l2*, (S–V) two independent *mllt10/af10* MOs, (W–Z) two independent *dot1l* MO, and (AA,AB) control MO. All MOs have been coinjected with an MO against *p53*. All images were captured using the same exposure and represent at least three independent experiments. In parentheses number of embryos showing described phenotype per number of total embryos analyzed.

Homozygous *apc* mutant zebrafish (*apc^mcr/mcr^*) embryos display a variety of developmental defects [41],[47],[48]. Overstimulation of the Wnt signaling pathway by abrogation of Apc function compromises intestinal development, as demonstrated by a failure to express the differentiation marker intestinal fatty acid binding protein (*i-fabp*) [39]. In these fish, the constitutive Wnt signal is thought to maintain an undifferentiated, proliferative state. Using a *tcf7l2* mutant zebrafish line, we showed recently that Tcf4 is a main effector of Wnt signaling during zebrafish intestine organogenesis and adult homeostasis [39],[40]. In agreement with these findings, injection of *tcf7l2* morpholino in *apc^mcr/mcr^* embryos rescued the expression of the intestinal differentiation marker *i-fabp*, as was observed previously using the *tcf7l2* mutant zebrafish line [39], at 80 hpf (Figure 8A–8D), while a control unrelated MO had no effect on *i-fabp* expression (Figure 8M and 8N) [39]. Consistent with the role of Mllt10/Af10-Dot1l as essential components of the activating Tcf4 complex, MO depletion of *mllt10/af10* (Figure 8E–8H) and *dot1l* (Figure 8I–8L) rescued *i-fabp* expression in *apc^mcr/mcr^* embryos. Other characteristic defects in *apc^mcr/mcr^* embryos are the upregulated mis-expression of *cyp26a1*, a major retinoic acid catabolic enzyme and a Wnt target gene [41],[47], as well as aberrant upregulation of *axin2*. Consistent with our observed rescue of *i-fabp* expression, depletion of *tcf7l2* (Figure 8O–8R, Figure S7A–S7D), *mllt10* (Figure 8S–8V, Figure S7E–S7H), and *dot1l* (Figure 8W–8Z, Figure S7I–S7L) rescued the mis-expression of both *cyp26a1* and *axin2* in *apc^mcr/mcr^* embryos. Thus, as with *tcf7l2*, the block in intestinal differentiation and the mis-expression of Wnt target genes in *apc^mcr/mcr^* embryos is rescued by depletion of *mllt10/af10* and *dot1l*, establishing these genes downstream of Apc as activators of Wnt target genes in vivo.

### *mllt10/af10* and *dot1l* Are Essential for Maintaining Intestinal Homeostasis during Zebrafish Development

**Figure 9:**
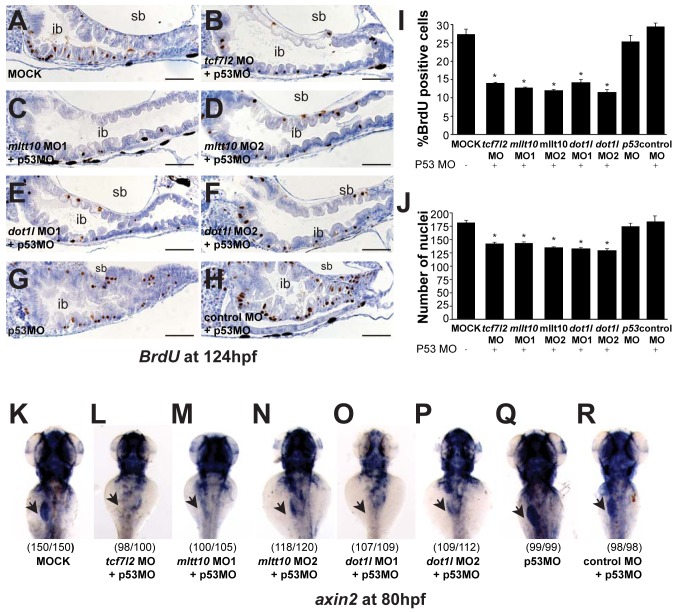
*tcf7l2*, *mllt10/af10*, and *dot1l* are essential for Wnt target gene expression and intestinal homeostasis in zebrafish. (A–H) Representative image of BrdU incorporation into epithelial cells in the intestinal bulb of 124 hpf zebrafish embryos. BrdU staining in wild-type embryos (A) and embryos depleted of *tcf7l2* (B), *mllt10/af10* (C,D), and *dot1l* (E,F), *p53* (G), and control (H) by MO injection. (I) Quantification (mean ± SD) of percent BrdU positive cells and (J) total number of cells in the epithelial layer of the intestinal bulb (20 embryos/condition). * *p*<0.05. Depletion of *tcf7l2*, *mllt10/af10*, and *dot1l* MO abrogates *axin2* expression in intestine. In situ hybridization for *axin2* in 80 hpf wild-type and MO-injected embryos (K–R). Staining for *axin2* in wild type embryos uninjected (K) or injected with MO against *tcf7l2* (L), *mllt10/af10* (M,N), and *dot1l* (O,P), *p53* (Q), and control MO (R). All MOs have been coinjected with a MO against *p53*. In parentheses number of embryos with phenotype per total analyzed. Images are representative of three independent experiments. sb, swim bladder; ib, intestinal bulb. Scale bar: 100 µm.

At 124 hpf the zebrafish intestinal tract can be divided into intestinal bulb, posterior intestine, and anal opening [49]. At this stage, the epithelial folding within the intestinal bulb is extensive and proliferation is restricted to cells at the base of the folds (Figure 9A, 9G, and 9H). To test whether proliferation in the intestinal epithelium is affected by depletion of *tcf7l2*, *mllt10/af10*, and *dot1l*, we injected MOs against these genes in one-cell stage embryos and performed bromodeoxyuridine (BrdU) incorporation analysis to mark cells progressing through the S-phase of the cell cycle at 124 hpf (Figure 9B–9H). First, to ensure that the expression of other *tcf/lef* family members is not affected upon MO depletion of *tcf7l2*, *mllt10/af10*, or *dot1l*, we performed RT-PCR analysis to check the expression levels of *lef1*, *tcf7*, *tcf7l1a*, and *tcf7l1b* (Figure S8). We found that MO depletion of *tcf7l2*, *mllt10/af10*, or *dot1l* had no effect on expression levels of the other *tcf/lef* family members. BrdU staining revealed a high ratio of proliferative BrdU-positive cells in the base of intestinal folds that was dramatically reduced following *tcf7l2* depletion (Figure 9B and 9I). We also observed a reduction in the total number of cells in the epithelial layer of the intestinal bulb, affecting the folding and the length of the villi (Figure 9J). The epithelial cells had a columnar shape and the cell nuclei were situated towards the base of the cells, suggesting that differentiation was not affected by depletion of *tcf7l2* (Figure 9B). In accordance with their role as transcriptional coactivators of Tcf4, depletion of *mllt10/af10* and *dot1l* significantly reduced the number of BrdU-positive cells and affected the formation of villi (Figure 9C–9J). We also analyzed the role of *tcf7l2*, *mllt10/af10*, and *dot1l* on transcription of *axin2*, a direct Wnt target gene in vivo. While expressed in the intestinal epithelium of embryos, either non-injected or injected with control MO at 80 hpf (Figure 9K, 9Q, and 9R), *axin2* expression was severely reduced in *tcf7l2*, *mllt10/af10*, and *dot1l* depleted morphants (Figure 9L–9P, and *apc^+/+^* embryos in Figure S7C–S7K).

Altogether, our results are consistent with the notion that *mllt10/af10-dot1l* may exert their activity through a specific functional interaction with tcf7l2 and not other *tcf/lef* proteins. Furthermore, our data reveal a critical role for Mllt10/Af10 and Dot1l in maintenance of intestinal homeostasis through their essential role as coactivators of the Tcf4/β-catenin complex in vivo.

## Discussion

The packaging of eukaryotic genomic DNA into chromatin provides a repressive environment for transcription. Activation of transcription requires the reorganization of chromatin into a more open structure, allowing access of sequence-specific transcription factors and the general transcription machinery. Previous studies have shown that to activate TCF target genes, β-catenin recruits chromatin-modifying and remodeling cofactors, which may work to generate a more open chromatin structure. Thus, studies have shown that β-catenin interacts with the chromatin remodeling factors BRG1 [20] and ISWI [21], the histone acetyltransferases p300/CBP [22],[23], and the H3K4 methyltransferase MLL [21]. We demonstrate the requirement of MLLT10/AF10-DOT1L and H3K79 methylation for Wnt target gene activation. Comparison of the differential gene expression profiles following depletion of MLLT10/AF10, DOT1L, as well as the previously known activators BRG1 and p300 reveals that in contrast to BRG1 and p300, which regulate a broad set of targets, most outside the Wnt pathway, over half of the differentially expressed genes upon depletion of MLLT10/AF10 or DOT1L are Wnt/β-catenin regulated targets. Thus, while BRG1 and p300 are pleiotropic activators in the Wnt as well as other pathways, MLL10 is more specific to Wnt target gene regulation than the other co-regulators tested.

We demonstrate the requirement of MLLT10/AF10-DOT1L and H3K79 methylation for Wnt target gene activation, specifically in the elongation step of transcription. While this manuscript was in preparation, Mohan and colleagues demonstrated the requirement of Dot1 and several Dot1 complex components in the regulation of a subset of wingless target genes in Drosophila [50]. Demonstrating epigenetic cross-talk, they also find histone H2B monoubiquitination by Bre1 to be a pre-requisite for bulk H3K79 trimethylation levels, required for the expression of a subset of wingless target genes [50].

Deciphering the co-factor requirement in the distinct steps of transcription activation will provide valuable mechanistic insight into how β-catenin co-ordinates the recruitment of this diverse series of chromatin modifiers to Wnt targets. An attractive model is the ordered cycling recruitment of chromatin modifying complexes, which are exchanged at the Wnt-responsive regulatory elements, in the distinct steps of transcription activation, ultimately leading to efficient RNA polymerase II-transcription initiation and elongation.

Our results support a model in which MLLT10/AF10 tethers the DOT1L methyltransferase to Wnt target genes. The restricted expression of *Mllt10/Af10* specifically in the mouse crypt proliferative compartment in the intestinal epithelium together with its temporal co-expression with *tcf7l2* in zebrafish embryos suggest a more specific role for Mllt10/Af10 in the regulation of Wnt target gene expression and as a recruiter of Dot1l to Wnt targets. Mllt10/Af10 appears to co-ordinate Dot1l activity with the β-catenin activating protein complex. Interestingly, our ChIP data indicate a distinct enrichment pattern between Mllt10/Af10 and Dot1l on Wnt target genes. While Mllt10/Af10 and β-catenin enrichment on Wnt target genes closely mirrors Tcf4, the recruitment of Dot1l and the resulting H3K79 di-/tri-methylation occurs over the entire target gene body and is essential for recruitment of elongating RNA Pol II to targets.

The mechanisms by which histone modifications introduced by Mllt10/Af10 actually lead to transcription activation are not well understood. Recent studies have shown that in mixed lineage leukemia, several MLL fusion partners, including MLLT10/AF10, may activate elongation of transcription by linking DOT1L to the pTEFb complex essential for transcription elongation [51]–[53]. In fact, DOT1L methylation of H3K79 was shown to be introduced during transcriptional elongation [32]. It is tempting to envision a mechanism whereby MLLT10/AF10 recruits DOT1L to the TCF4/β-catenin complex, which contains coactivators that stimulate the initiation phase of transcription. Upon MLLT10/AF10 mediated recruitment to TCF4/β-catenin target genes, DOT1L is loaded onto the elongating RNA pol II complex to facilitate transcription elongation.

We demonstrate the in vivo physiological role of Mllt10/Af10-Dot1l in intestinal development and homeostasis using zebrafish as a model system. Depletion of Tcf4, Mllt10, and Dot1l results in a significant reduction in the number of BrdU stained proliferating intestinal cells in the intervillus junctions of zebrafish embryos. The high rate of intestinal cell proliferation determines the formation of intestinal invaginations, essential for the physiological function of the intestine. Reduction in proliferation in the intestinal epithelium as a consequence of depletion of Mllt10/Af10 or Dot1l demonstrates the relevance of these coactivators in the Wnt dependent maintenance of intestinal structure and function.

Abrogation of Apc function during zebrafish development arrests affected cells in a proliferative state, likely due to inappropriate activation of Tcf target genes, leading to a blockage in differentiation of the intestinal epithelium [39],[47]. We previously showed using a Tcf4 mutant line crossed with *apc^mcr/mcr^* that although Tcf4 depletion rescues some of the intestinal differentiation defects in *apc^mcr/mcr^* fish, it does not rescue the *apc^mcr/mcr^* defect in valve formation, blood circulation, and as a consequence, the survival of *apc^mcr/mcr^* fish [39]. Consistent with those observations, MO depletion of *tcf7l2*, *mllt10*, and *dot1l* does not rescue these defects in *apc^mcr/mcr^* fish (unpublished data). Despite the inability to revert all of the *apc^mcr/mcr^* defects, MO depletion of Tcf4, Mllt10, and Dot1l in *apc^mcr/mcr^* fish rescues the block in intestinal differentiation as well as the mis-expression of the Wnt target genes *cyp26a1* and *axin2*. Thus, Tcf4 plays a crucial role during intestinal development and acts as the main downstream effector of the Wnt pathway in this tissue [39]. We present evidence that Mllt10/Af10-Dot1l are part of the β-catenin/Tcf4-dependent transcriptional complex and that these coactivators also play an important role in the organogenesis of the zebrafish intestine.

Our data are consistent with *tcf7l2* as the principle *tcf/lef* family member involved in expression of *axin2* in the intestinal epithelium and GFP in the hindbrain in wild type and TOPdGFP line, respectively. We observe similar down-regulation of GFP expression in the TOPdGFP line as well as down-regulation of the target gene *axin2* also upon depletion of *mllt10* and *dot1l*. While our data suggest that the expression of *axin2* in the intestinal epithelium of wild-type embryos and GFP in the hindbrain of TOPdGFP line is highly depended on *tcf7l2*, we cannot exclude possible redundant regulation of other developmentally important Wnt-targets by other tcf/lef members. The observed residual expression of GFP in the TOPdGFP Tcf4, Mllt10, and Dot1l morphant embryos may reflect the role of other *tcf/lef* members in controlling the expression of Tcf/Lef-derived genes in the hindbrain.

We have previously published that the *tcf7l2* mutant zebrafish line does not show any strong morphological defects during early embryogenesis [39],[40]. Consistent with this observation, our MO depletion of *tcf7l2* mimics the absence of morphological defects during early embryogenesis. The overall lack of early macroscopic morphological defects upon *mllt10* and *dot1l* depletion together with their requirement for intestinal homeostasis is consistent with their specific functional dependence on *tcf7l2* and not other tcf/lef family members (such as the headless phenotype of *tcf7l1a* depleted embryos [39],[54] or the macroscopic morphological defects resulting from depletion of lef1 [43],[55]; unpublished data). Additionally, contrary to Tcf4, Mllt10, and Dot1l depleted embryos, depletion of Tcf7 [39],Tcf7l1a [39], and Lef1 (unpublished data) does not rescue any of the intestinal differentiation defects, such as absence of *i-fabp* expression or mis-expression of *cyp26a1* or *axin2* in the *apc^mcr/mcr^* line. Thus, altogether, our results suggest that Mllt10-Dot1l exert their activity through Tcf4 and not other Tcf/Lef family members. Future studies will determine the mechanisms underlying the observed specificity of Mllt10 and Dot1l for the Wnt response downstream of Tcf4 as opposed to other TCF/LEF members. Whether this specificity arises merely from the co-expression of these genes in the tissues affected or whether the formation of a specific Mllt10-Dot1l complex with β-catenin and TCF4 but not the other family members mediates a specific response on TCF4 target genes are possibilities that remain to be examined.

Although MLLT10/AF10 contains structural motifs indicative of chromatin-associated functions, its biological role has remained unclear. Our results provide insight into the physiological role of MLLT10/AF10 as a critical coactivator of Wnt target genes essential for transcription elongation. Thus far, studies on mammalian MLLT10/AF10 have focused on its function as a fusion partner of MLL or CALM in leukemia. Given the involvement of MLLT10/AF10 in Wnt target gene activation, it will be interesting to probe potential de-regulation of Wnt signaling as a consequence of the MLLT10/AF10-MLL/CALM translocation in leukemia.

Future studies will illuminate the complex interplay of interactions between β-catenin/TCF4 and the various chromatin modifying and associated cofactors, leading to regulation of Wnt targets in normal intestinal development and tumorigenesis. The discovery of novel enzymatic activities that are essential and dedicated to Wnt target gene activation, such as DOT1L, presents potential new targets for rational drug development for the treatment of cancer.

## Materials and Methods

### Isolation of Mouse Crypt and Villus Fractions

For a detailed description of the biochemical fractionation method of mouse crypt and villus purification, please refer to [25]. Briefly, mouse small intestine was washed with PBS and cut open longitudinally and into small 1–2 cm pieces. Crypt and villus fractions were isolated using several rounds of incubations in mild PBS-EDTA/EGTA chelation solution combined with vigorous shaking followed by further purification using 70 um (for villi) and 40 um (for crypts) cell strainers. All solutions and incubations were carried out at 4°C.

### Immunoprecipitation

Total cellular extracts from Ls174T CRC cells or primary mouse crypt or villus material were prepared in PLB buffer (1% TritonX-100, 2 mM EDTA, 1 mM DTT in PBS) supplemented with protease inhibitors (Complete, Roche Molecular Biochemicals). Cellular lysates were precleared with IgG-agarose beads (Sigma) for 6 h at 4°C. Immunoprecipitations of endogenous complexes from mouse crypt, villus, and Ls174T cells were carried out overnight at 4°C, with anti-TCF4 (Santa Cruz), anti β-catenin (BD transduction), anti-Mllt10/Af10 (Santa Cruz or abcam), or anti-DOT1L (Abgent) antibodies at concentrations of 2 µg/mL in combination with a 50% protein G-Agarose slurry (Sigma). Immunoprecipitated material was washed four times in PLB buffer. Bound proteins were subjected to SDS-PAGE and Western blot analysis or Mass Spectrometry Analysis. Western blot analysis was also conducted using antibodies against TCF-1 (upstate), EPHB2 (Abgent), and p300 and BRG1 (SantaCruz).

### Mass Spectrometry Analysis

For details of the mass spectrometry analysis, please see [25]. Briefly, gel bands containing Mllt10/Af10 and Dot1l were subjected to in-gel digestion with trypsin overnight at 37°C. Peptides were extracted and subjected to nanoscale liquid chromatography tandem mass spectrometry (nanoLC-MS/MS) analysis, performed on an Agilent 1100 HPLC system (Agilent technologies) connected to an LTQ Linear Ion Trap Mass Spectrometer combined with either an Orbitrap (ThermoFisher, Waltham, MA) or an Fourier Transform Ion Cyclotron Resonance cell (ThermoFisher, Waltham, MA). For protein identification, raw MS data were converted to peak lists using Bioworks Browser software, version 3.1.1. Spectra were searched against the IPI (International Protein Index) mouse database version 3.36 (51,326 sequences; 23,682,061 residues) using Mascot software version 2.2.0 (www.matrixscience.com), with trypsin set as enzyme.

### Histology, Immunohistochemistry, and Confocal Microscopy

Mouse small intestine tissue was fixed in 10% formalin, paraffin embedded, and sectioned. Human colon samples were obtained immediately after resection. The tissue was fixed in 4.0% formaldehyde in PBS for 2 h at 4°C. Tissues were cryo protected in 30% Sucrose o/n at 4°C after which samples were frozen in Tissue Tec. Sections of 16 um were made with a Leica CryoStat. Sections were immunostained after permeabilization with 0.5% Triton X-100 in 1% BSA, 1% Glycine, 0.1% Lysine in PBS. Immunostaining was done in the permeabilization buffer with Mllt10/Af10 (Santa Cruz) and E-Cadherin (Transduction Laboratories) antisera o/n at 4°C. Secondary antibody (InVitrogen Alexa 488 and 568) staining was done for 1 h at 4°C. Fluorescent imaging was done with a Leica confocal laser scanning microscope.

For histology, 124 hpf zebrafish embryos were fixed in 4% (w = v) paraformaldehyde in PBS overnight at 4°C with agitation, rinsed in PBS, dehydrated through a standard ethanol series to 100%, embedded in paraffin, and sectioned at 6 µm intervals for staining.

GFP imaging in TOPdGFP zebrafish line was performed using a Leica SP5 confocal microscope and images were processed with Leica Application Suite.

### RNA Interference

Pre-designed siRNA pools targeting transcripts of the human β-catenin (L-003482-00), MLLT10/AF10 (L-019827-00), DOT1L (L-014900-01), BRG1 (SMARCA4) (L-010431-00), p300 (L-003486-00), and non-target control siRNA pool (D-001810-10-20) were purchased from Dharmacon and used to knock down the corresponding genes in Ls174T CRC and HEK293T cells. siRNA was delivered into Ls174T and HEK293T cells using either the siLentFect Lipid Reagent (BioRad) or INTERFERin reagent (Polyplus Transfections). siRNA (15 nM) was used to transfect 2×10^5^ cells, and protein levels were examined by Western blot analysis 72–96 h after transfection.

### Luciferase Assays

Ls174T CRC cells containing integrated TOPFLASH and FOPFLASH (optimal and mutated TCF luciferase-reporter constructs as described previously) [5] were transfected with control non-targeting siRNA or siRNA targeting the human MLLT10/AF10 or β-catenin. After 72–96 h, the cells were lysed in luciferase lysis buffer (Promega Corp.), and luciferase activities were measured using the Dual-Luciferase reporter assay system. The data shown are of two independent triplicate experiments as the mean ± S.D. of the relative light units.

### ChIP

ChIP was performed on Ls174T CRC and HEK293T cells or purified mouse crypt and villus fractions as previously described [25]. 2–5 µg of the antibodies TCF4 (Santa Cruz), β-catenin (BD Transduction), MLLT10/AF10 (Santa Cruz and abcam), DOT1L (Abgent), H3K79 dimethyl, H3K79 trimethyl, H3K4 trimethyl (upstate), RNA PolII (Santa Cruz), RNA PolII phos Ser2 (H5), and RNA PolII phos Ser5 (H14, Covance) was incubated with the sheared cross-linked chromatin to immunoprecipitate the indicated complexes. Input and immunoprecipitated DNA were subjected to Sybergreen Q PCR cycles with primers overlapping the gene body and upstream and downstream regulatory regions of mouse and human *AXIN2*, *c-MYC*, and *ZCCHC12* genes.

### Microarray Analysis

Ls174T and HEK293T cells were transfected with siRNA (si β-catenin, si MLLT10/AF10, si BRG1, si p300, and si Control). HEK293T cells were then induced with Wnt3A or control medium to measure direct Wnt regulated activity. Total RNA was extracted from samples using RNeasy Mini Kit (Qiagen). 2 µg of RNA from each sample, together with universal human reference RNA (Strategene), was labeled by Quick Amp Labelling Kit, two color (Agilent) with Cy3 and Cy5, respectively. Samples were hybridized to 4×44K Whole Human Genome dual color Microarrays (Agilent, G4112F). Microarray signal and background information were retrieved using Feature Extraction program (V.9.5.3, Agilent Technologies). For each pair of experiments, fluorescent signals in either channel with greater than 2-fold above the local background were considered as well-measured. Corresponding fold changes of Ls174T cells after siβ-catenin, si MLLT10/AF10, si BRG1, or si p300 suppression were calculated by normalizing with the siControl samples. In HEK293T cells, Wnt regulated genes after 9 h Wnt induction were obtained by normalizing the fold change between 9 h Wnt3A and Control medium induction in siControl samples (no Wnt), and the corresponding fold changes of si MLLT10/AF10, si DOT1L, si BRG1, and si p300 upon 9 h Wnt induction were obtained by normalizing with siControl samples after 9 h Wnt induction. The raw data were submitted to Gene Expression Omnibus, accession number GSE21367.

### qPCR

ChIP experiments were analyzed by qPCR in an iCycler iQ real-time PCR detection system (BioRad) using iQ Sybergreen Supermix (BioRad). ChIP values were normalized as a percentage of input and presented as the percentage of input immunoprecipitated. Primer pairs spanning the upstream regulatory regions, gene body, and downstream regulatory regions of mouse *Axin2* and *c-Myc* genes and human *AXIN2*, *c-MYC*, and *ZCCHC12* genes were used in qPCR analysis. Primer sequences are provided in Table S4.

### Zebrafish Husbandry

Zebrafish were maintained on a 14-h-light/10-h-dark cycle at 28.5°C. Fertilized embryos were collected after natural spawning, cultured, and staged by developmental time and morphological criteria. *apc^mcr^* and *TOPdGFP* lines were previously described [44],[48]. *apc^mcr^* mutants were genotyped as described [48].

### Computer Sequence Analysis

*Drosophila melanogaster*, *Homo sapiens*, *Mus musculus*, and trace cDNA sequences for *Danio rerio mllt10/af10* were analyzed with ENSEMBL. Full exon-intron structures of *mllt10/af10 danio rerio* prediction were performed with Genome Scan. Phylogenetic tree of *Mllt10/Af10* and domain alignment were performed with CLUSTAL V (DNAstar).

### Expression Analyses

Embryos at different developmental stages were homogenized in TRIzol reagent (Invitrogen) using a homogenizer. Total RNA was isolated according to the manufacturer's instructions. Single-stranded cDNA was synthesized from 1 µg total RNA using Superscript III (Invitrogen). PCR primers used (Table S4) were designed spanning an intron. PCRs were performed in triplicate; *n*>50 embryos.

RT-PCR primers used to detect mRNA expression of Wnt target genes human *c-MYC*, *AXIN2*, *LGR5*, *EPHB3*, and *ASCL2* in Ls174T CRC are provided in Table S4.

RT-PCR primers used to detect mRNA expression of zebrafish genes *tcf7l2*, *mllt10*, *dot1l*, *tbp*, *tcf7*, *tcf7l1a*, *tcf7l1b*, and *lef1* are provided in Table S4.

### Morpholino Microinjections

Splicing morpholino oligonucleotides for *tcf7l2*, *mllt10*, and *dot1l* were designed in house and obtained from Gene Tools LLC (OR, USA). The blocking MO against p53 has been previously described (Table S4) [45]. For microinjections, 2 ng MO were injected into wild-type TL, TOPdGFP, and *apc^mcr^* line embryos at one-cell stage. In co-injection experiments, we used 1 ng of *p53* MO and 2 ng of the corresponding MO. Injection experiments were performed in triplicate; in each replica *n*>100 embryos were analyzed.

### Whole-Mount In Situ Hybridizations

Embryos were collected at 56, 72, and 80 hpf, fixed in 4% paraformaldehyde in PBS overnight at 4°C, rinsed in PBS, dehydrated through a standard methanol series to 100%, and stored at −20°C. Whole-mount in situ hybridizations (WISH) were carried out as described [56]. Digoxigenin-labeled riboprobes were generated by linearization of pGEM (Promega) containing a fragment of *tcf7l2*, *mllt10/af10* (two different sequences), *dot1*l (two different sequences), *dGFP*, *i-fabp*, *cyp26a1*, or *axin2* cDNA followed by in vitro transcription and purified using NucleoSpin RNA clean-up columns (Machery-Nagel). Pictures were obtained using a Leica microscope (TCS NT), digitized with a camera (DFC480; Leica), and processed with IM500 Image Manager (Leica). WISH was preformed for *i-fabp* in *apc^mcr^/^mcr^* embryos after MO injection, expression scored, and each embryo genotyped. Experiments were in triplicate; in each replica, *n*>100 embryos were analyzed.

### Detection of Proliferating Cells

122 hpf live embryos (previously MO injected at one-cell stage) were immersed in embryo medium with BrdU (Sigma) at 3 mg/mL for 30 min at 28.5°C. After several washes with pre-warmed embryo medium, embryos were left for 1 h at 28.5°C in embryo medium, euthanized, and fixed in 4% paraformaldehyde in PBS overnight at 4°C. Detection of BrdU was carried out by immunostaining with a mouse anti-BrdU antibody (Dako, Glostrup).

## Supporting Information

Figure S1 **Peptides identified and coverage of (A) Mllt10 and (B) Dot1l in Tcf4 complex in mouse small intestinal crypt.** Amino acid sequences of Mllt10 and Dot1l detected in the Tcf4 immunoprecipitate from crypt lysates are underlined.
(0.03 MB PDF)

Figure S2 **Specific enrichment of Mllt10 and Dot1l, and H3K79 di-/tri-methylation at** ***c-Myc*** **locus in mouse crypts.** (A) Schematic representation of the mouse amplicons scanned in ChIP experiments by qPCR. Purified crypt and villus fractions from mouse intestine were subjected to ChIP using antibodies directed against Tcf4 (B), β-catenin (C), Mllt10/Af10 (D), Dot1l (E), di-methyl H3K79 (F), and tri-methyl H3K79 (G). Chromatin was immunoprecipitated with the specified antibodies followed by qPCR using primer pairs spanning the *c-Myc* locus as indicated in (A). Results are presented as percent immunoprecipitated over input and are representative of three independent experiments.
(0.07 MB PDF)

Figure S3 **β-catenin recruits Mllt10/Af10 to Wnt targets.** (A) Ls174T CRC lysates were immunoprecipitated with antibodies against TCF4 (left panel) and β-catenin (right panel) and analyzed by Western blotting with the indicated antibodies for binding to MLLT10/Af10 and DOT1L. (B–H) β-catenin-dependent H3K79 methylation and recruitment of MLLT10 and DOT1L to *c-MYC* gene in Ls174T CRC. (B) Schematic representation of human *c-MYC* locus and amplicons scanned in ChIP assays by qPCR. ChIP experiments in Ls174T CRC uninduced or induced with Dox using antibodies against (C) TCF4, (D) β-catenin, (E) MLLT10, (F) DOT1L, (G) H3K79 dimethyl, and (H) H3K79 trimethyl. Immunoprecipitated DNA was analyzed by qPCR using primer pairs specific for the *c-MYC* locus as indicated. Results are presented as percent immunoprecipitated over input and are representative of three independent experiments.
(0.08 MB PDF)

Figure S4 **Mllt10/Af10 interacts directly with β-catenin.** (A) Recombinant GST-fused TCF4 and β-catenin proteins were used in pull-down assays with in vitro translated S35 labeled MLLT10 to examine direct interaction. (B) Schematic representation of N-Terminal and C-Terminal MLLT10 deletion mutants and S35 labeled ΔC- and C-Terminal β-catenin deletion mutants used in GST pulldown assays (C). N-Terminal MLLT10 interacts directly with the β-catenin C-terminal domain.
(0.03 MB PDF)

Figure S5 (A) Significant overlap between differentially regulated genes in response to MLLT10 or DOT1L depletion in HEK293T cells after Wnt stimulation. Comparison of the corresponding expression pattern after siRNA suppression of MLLT10/AF10 or DOT1L in Wnt induced condition. Heatmap showing 1,116 transcripts after siRNA depletion of MLLT10 and 9 h Wnt stimulation in HEK293T cells with greater than 1.5-fold variation. Also shown is comparison of the corresponding expression pattern of genes induced after 9 h Wnt treatment and after siRNA suppression of DOT1L. Red, upregulated after MLLT10 suppression; green, downregulated after MLLT10 suppression; grey, missing data. (B) Venn diagram comparatively depicting genes suppressed upon MLLT10 and DOT1L depletion in HEK293T cells under Wnt-induced conditions.
(0.03 MB PDF)

Figure S6 **mRNA expression of** ***tcf7l2*** **,** ***mllt10*** **, and** ***dot1l*** **during zebrafish embryonic development and their depletion by splice-inhibiting MO sequences.** (A) Phylogenetic tree analysis places zebrafish *(Danio rerio) Mllt10* close to mouse and human sequences. (B–C) Amino acid sequence alignment of the Leucine zipper and PHD finger domains show high conservation of *Mllt10* between species. (D) RT-PCR analysis of *tcf7l2*, *mllt10*, *dot1l*, and *tbp* RNA expression levels in whole embryos at different stages of embryonic development. (E) Representation of the splice-blocking MO sequences (blue) generated to deplete mRNA levels for *tcf7l2*, *mllt10*, and *dot1l*.
(0.03 MB PDF)

Figure S7 **Depletion of** ***tcf7l2, mllt10/af10*** **and** ***dot1l*** **rescues mis-expression of** ***axin2*** **in apc^mcr/mcr^ zebrafish, placing these genes downstream of Apc as Wnt target gene activators (A–N).** Representative whole mount in situ hybridizations for *axin2* in wild type and *apc^mcr/mcr^* mutant embryos at 80 hpf injected with (A,B) buffer alone, (C,D) MO against *tcf7l2*, (E–H) two independent *mllt10/af10* MOs, (I–L) two independent *dot1l* MO, and (M,N) control MO. All MOs have been coinjected with a MO against *p53*. All images were captured using the same exposure and represent at least three independent experiments. In parentheses number of embryos showing described phenotype per number of total embryos analyzed.
(0.06 MB PDF)

Figure S8 **Depletion of** ***tcf7l2*** **,** ***mllt10*** **, or** ***dot1l*** **does not affect the expression levels of other** ***tcf/lef*** **family members in zebrafish embryos.** RT-PCR analysis of *lef1*, *tcf7*, *tcf7l1a*, *tcf7l1b*, and *tbp* RNA expression levels in whole embryos injected with MO against *p53* alone, or MO against *p53* coinjected with MOs against *tcf7l2*, *mllt10*, *dot1l*, or control MO, respectively.
(0.02 MB PDF)

Table S1 **List of proteins with highest scores identified by MS in Tcf4 crypt complex.**
(0.03 MB XLS)

Table S2 **Ls174T Wnt regulated gene list—1,003 transcripts.**
(0.24 MB XLS)

Table S3 **HEK293T Wnt-induced gene list after 9 h Wnt induction—1,189 transcripts.**
(0.30 MB XLS)

Table S4 **Primer and Morpholino sequences.**
(0.03 MB XLS)

## Abbreviations

BrdU: bromodeoxyuridine
CALM: clathrin-assembly protein-like lymphoid-myeloid
ChIP: chromatin immunoprecipitation
hpf: hours post-fertilization
*i-fabp*: intestinal fatty acid binding protein
qPCR: quantitative PCR
