# Correction: The Leukemia-Associated Mllt10/Af10-Dot1l Are Tcf4/β-Catenin Coactivators Essential for Intestinal Homeostasis

**DOI:** 10.1371/journal.pbio.1002596

**Published:** 2017-02-07

**Authors:** Tokameh Mahmoudi, Sylvia F. Boj, Pantelis Hatzis, Vivian S. W. Li, Nadia Taouatas, Robert G. J. Vries, Hans Teunissen, Harry Begthel, Jeroen Korving, Shabaz Mohammed, Albert J. R. Heck, Hans Clevers

## Notice of republication

Due to the publication of copyrighted material, this article has been republished in order to remove dual published content from the published record. Please see below for full details.

The authors would like to make the following corrections to four of the published figures–Figs 1, 3, 5 and 6. The editors have verified all relevant original data and also confirm that the manuscript’s original conclusions are not affected. The authors apologize for these errors caused by mislabelling and inadvertent duplication during initial manuscript preparation. All authors have seen and approved this republication.

**Fig 1:**
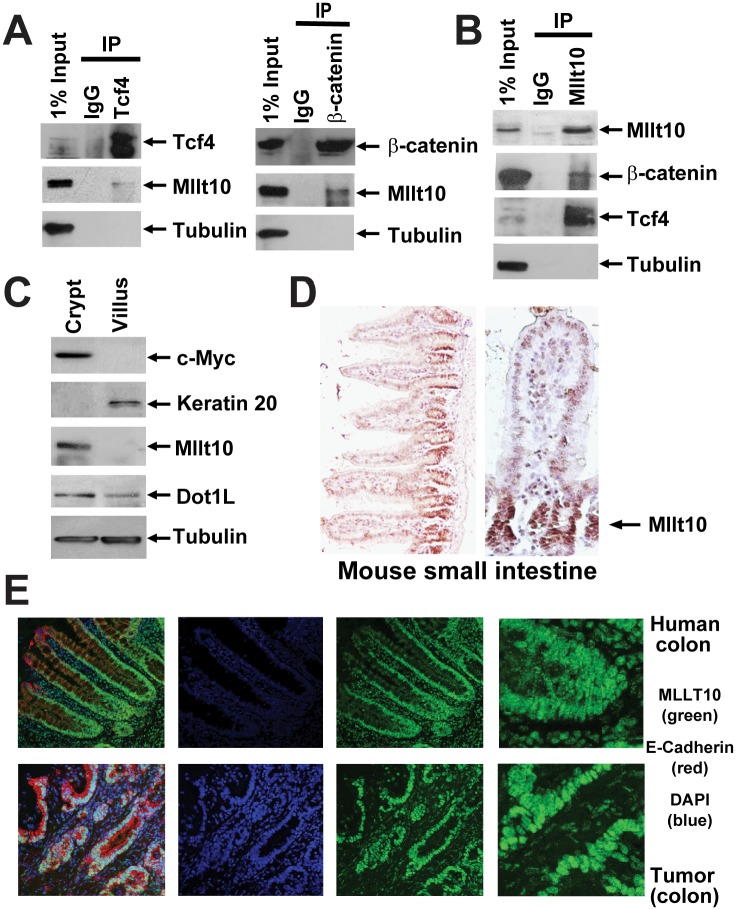
Mllt10/Af10 co-immunoprecipitates with Tcf4 in the mouse small intestinal crypt. (A) Cell lysates from purified crypt fractions were immunoprecipitated with antibodies directed against endogenous Tcf4, β-catenin, and (B) Mllt10/Af10 as indicated and analyzed by Western blotting with the indicated antibodies. (C) Western blot analysis of mechanically fractionated crypt and villus from mouse small intestine using antibodies directed against a gene expressed specifically in the crypt (c-Myc), villus (Keratin 20), Tubulin as control, as well as Mllt10/Af10 and Dot1l. (D) Mllt10/Af10 antibody staining on mouse small intestinal epithelium. Arrow indicates expression in the crypt proliferative compartment. (E) Confocal image for MLLT10/AF10 (green), E-Cadherin (red), and the nuclei counter stained with DAPi (blue). MLLT10/AF10 is expressed in nuclei of normal colon epithelium in a gradient concentrated at the crypt bottom and in colorectal cancer cells.

**Fig 3:**
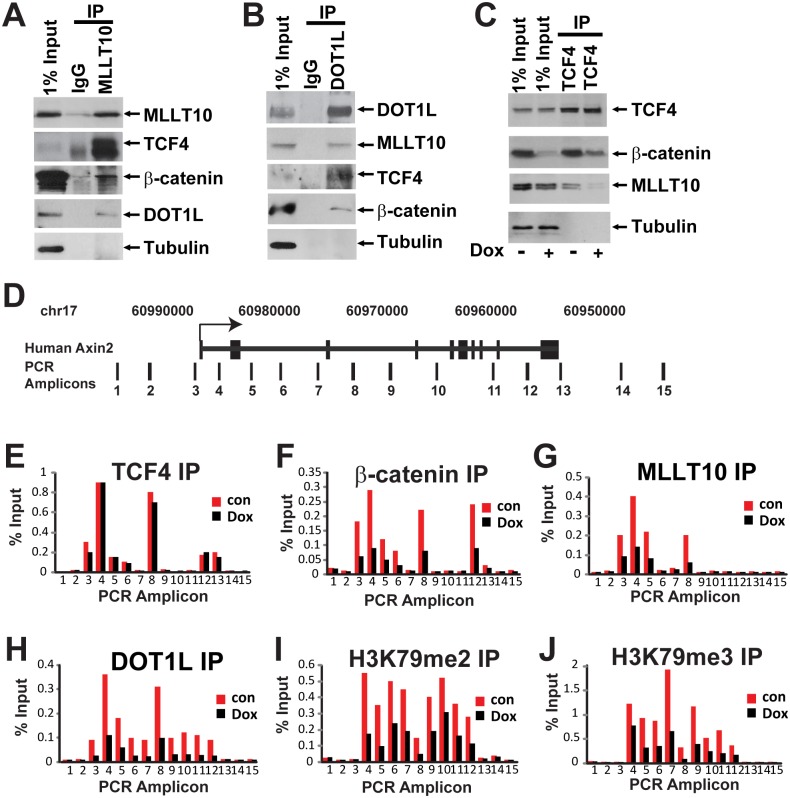
β-catenin-dependent H3K79 methylation and MLLT10/AF10, DOT1L recruitment to human *AXIN2* gene. Cell lysates from Ls174T CRCs were immunoprecipitated with antibodies against endogenous MLLT10/AF10 (A) and DOT1L (B) complexes and analyzed by Western blotting with the indicated antibodies. (C) MLLT10/AF10 interaction with TCF4 is mediated by β-catenin. Western blot analysis of β-catenin depletion in Ls174T cells expressing doxycycline (Dox)-inducible β-catenin shRNA. Immunoprecipitated TCF4-protein complexes from untreated or Dox-treated cells were resolved by SDS-PAGE followed by Western blotting with the indicated antibodies. (D) Schematic representation of the human *AXIN2* locus and amplicons scanned in Chromatin immunoprecipitation experiments by qPCR. ChIP in Ls174T CRCs uninduced or induced with Dox using antibodies specific for TCF4 (E), β-catenin (F), MLLT10/AF10 (G), DOT1L (H), H3K79 dimethyl (I), and H3K79 trimethyl (J). The immunoprecipitated DNA was analyzed by qPCR using primers specific for the *AXIN2* locus as indicated. Results are presented as percent immunoprecipitated over input and are representative of three independent experiments.

**Fig 5:**
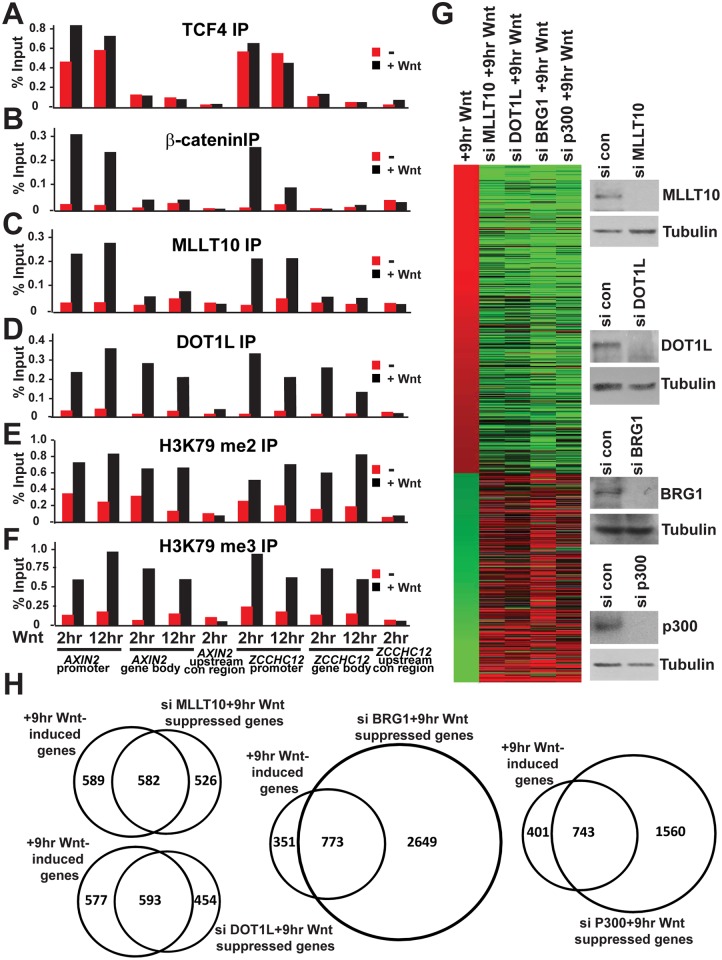
MLLT10/AF10 and DOT1L are essential and dedicated to the Wnt-induced transcriptional program in HEK293T cells. Wnt-induced association of MLLT10/AF10-DOT1L with and regulation of Wnt target genes in HEK293T cells. (A–F) ChIP assays in HEK293T cells uninduced or induced with Wnt3A conditioned media at 2 and 12 h using antibodies specific for TCF4 (A), β-catenin (B), MLLT10/AF10 (C), DOT1L (D), H3K79 di-methyl (E), and H3K79 tri-methyl (F). The immunoprecipitated DNA was analyzed by qPCR using primers specific for the *c-MYC* and *ZCCHC12* loci as indicated. Results are presented as percent immunoprecipitated over input and are representative of three independent experiments. (G) Comparison of the corresponding expression pattern after siRNA suppression of MLLT10/AF10, DOT1L, BRG1, and p300 in the Wnt induced condition. Heatmap showing 1988 Wnt regulated transcripts after 9 h Wnt-induction (relative to uninduced sample (no Wnt)) in HEK293T cells with greater than 1.5-fold variation, and the comparison of the corresponding expression pattern after siRNA suppression of MLLT10/AF10, DOT1L, BRG1, and p300 in Wnt-induced condition (relative to 9 h Wnt induction). Red, upregulated after Wnt; green, downregulated after Wnt induction; grey, missing data. Western blot analysis of MLLT10/AF10, DOT1L, BRG1, p300, and Tubulin upon siRNA depletion of each gene as indicated. (H) Venn diagram depicting the comparison of Wnt-induced genes and genes downregulated after MLLT10/AF10, DOT1L, BRG1, or p300 suppression in HEK293T cells after Wnt induction.

**Fig 6:**
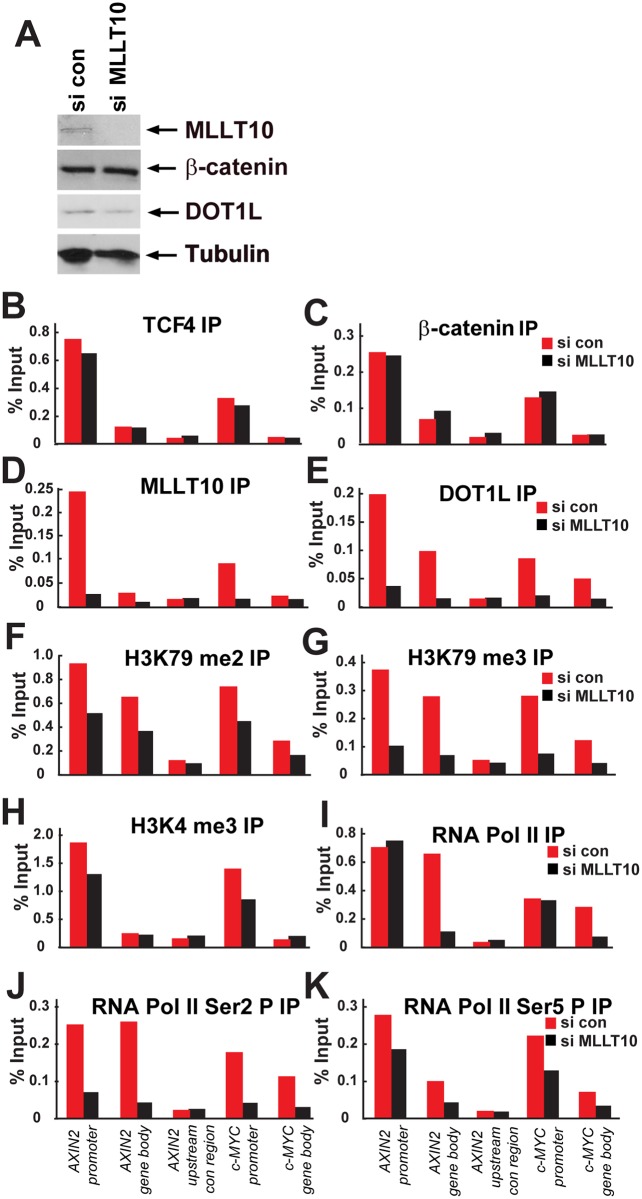
MLLT10/AF10 targets DOT1L-H3K79 methylation to Wnt target genes and is essential for transcription elongation. (A) Expression levels of MLLT10/AF10, β-catenin, DOT1L, and Tubulin analyzed by Western blotting after siRNA depletion of MLLT10/AF10. ChIP experiments in Ls174T CRCs containing or depleted of MLLT10/AF10 by siRNA using antibodies against (B) TCF4, (C) β-catenin, (D) MLLT10/AF10, (E) DOT1L, (F) H3K79 dimethyl, (G) H3K79 trimethyl, (H) H3K4trimethyl, (I) RNA Pol II, (J) RNA Pol II Ser2P, and (K) RNA Pol II Ser5P. Immunoprecipitated DNA was analyzed by qPCR using primers specific for *AXIN2* and *c-MYC* promoters and unbound *AXIN2* upstream control region. Results are presented as percent immunoprecipitated over input and are representative of three independent experiments.

### Corrections to Fig 1

The originally published version of Fig 1C included a Tubulin (crypt-villus) panel had been previously published in Fig 1A of a publication in another journal (The EMBO Journal (2009) 28, 3329–3340). Both panels are correct, but we are re-publishing Fig 1 in this *PLOS Biology* article to include a replicate of the Tubulin (crypt-villus) panel in Fig 1C to avoid issues of copyrighted dual publication. The corrected Fig 1 file is provided.

### Corrections to Fig 3

The published Fig 3A MLLT10 bands are incorrect; an MLLT10 panel from Fig 4B was accidentally duplicated in its place. The correct MLLT10 bands are now provided in this revised figure.

The published Fig 3A TCF4 bands are incorrect; a similar-looking beta-catenin panel from Fig 1B was accidentally duplicated in its place. The correct TCF4 bands are now provided in this revised figure.

The published Fig 3B Tubulin panel is incorrect; a similar-looking Tubulin panel from Fig 1A was accidentally duplicated in its place. The correct Tubulin bands are now provided in this revised figure.

The published Fig 3B MLLT10 panel is incorrect; a DOT1L panel from a beta-catenin IP experiment in Fig S3A was accidentally duplicated in its place. The correct MLLT10 bands are now provided in this revised figure.

### Correction to Fig 5

Fig 5G Tubulin panel (for si con, si DOT1L) is incorrect; a similar-looking DOT1L panel from Fig 4A was accidentally duplicated in its place. The correct Tubulin bands are now provided in this revised figure.

### Corrections to Fig 6

The originally published version of Fig 6A included a beta-catenin (si MLLT10) panel that we had previously published in Fig 5B (as beta-catenin, si TNIK) of a publication in another journal (The EMBO Journal (2009) 28, 3329–3340). The EMBO publication’s panel is correct; this *PLOS Biology* article’s panel was incorrect. We have now corrected Fig 6 to include the correct beta-catenin (si MLLT10) panel in Fig 6A. We are also re-publishing this corrected Fig 6 to avoid issues of incorrect dual publication. The corrected Fig 6 file is provided.
