# Supplementary material for: The Leukemia-Associated Mllt10/Af10-Dot1l Are Tcf4/β-Catenin Coactivators Essential for Intestinal Homeostasis
Source: PLoS Biol. 2010 Nov 16;8(11):e1000539. doi: 10.1371/journal.pbio.1000539 (PMC2982801; doi:10.1371/journal.pbio.1000539)
Supplement: Figure S1 — Peptides identified and coverage of (A) Mllt10 and (B) Dot1l in Tcf4 complex in mouse small intestinal crypt. Amino acid sequences of Mllt10 and Dot1l detected in the Tcf4 immunoprecipitate from crypt lysates are underlined. (0.03 MB PDF) [file pbio.1000539.s001.pdf]

A

Mllt10 Myeloid/lymphoid or mixed lineage-leukemia translocation to 10 homolog
Mass: 114157 Score: 109 Queries matched: 3 emPAI: 0.07

Query	Observed	Mr(expt)	Mr(calc)	ppm	Miss	Score	Expect	Rank	Peptide
394	458.2669	914.5193	914.5185	0.84	1	32	0.046	1	RRLEEQIK.N
428	466.2518	930.4890	930.4883	0.77	1	50	0.00066	1	K.RLEDTAAR.F
1992	780.3823	1558.7500	1558.7475	1.61	0	84	1.7e-007	1	K.TYTSTSNNSISGSLK.N

MVSSDRPVSLEDEVSHSMKEMIGGCCVCSDERGWAENPLVYCDGHGCSVAVHQACYGIVQVPTGPWFCRKCESQ
AARVRCELCPHKDGALKRTDNGGWAHVVCALYIPEVQFANVSTMEPIVLQSVPHDRYNKTCYICDEQGRESKAATG
CMTCNKHGCRQAFHVTCAQFAGLLCEEEGNGADNVQYCGYCKYHFSKLKKSKRGSNRSYDQSLSDSSSHSQDKHHE
EKKKYKEKDKHKQKHKKQPEPSPALVPSLTVTTEKTYTSTSNNSISGSLKRLEDTAARFTNANFQEVSAHTSSGKDVSETR
GSEGKGKKSSAHSSGQRGRKPGGGRNPGTTVSAASPFPQGSFSGTPGSVKSSSGSSVQSPQDFLSFTDSDLRNDSYSH
QQSSATKDVHKGESGSQEGGVNSESTLIGLPSTSAVTSQPKSFENSPGDLGNSSLPTAGYKRAQTSGIEEETVKEKKRK
NKQSKHGPGRPKGNKNQENVSHLSVSSAPSTSSVASAAGSITSSSLQKSPTLLRNGSLQSLSVGSSPVGSEISMQYRHD
ACPTTTFSELLNAIHNDRGDSSTLTKQELKFIGIYNSMDVAVSFPNVVSGSGSSTPVSSSHLPQQSSGHLQQVGALSPSA
SSAAPAVATTQANTLSGSSLSQAPSHMYGNRSNSSMAALIQSENNQTDQDLGDNSRNLVGRGSSPRGSLSPRSPVS
LQIRYDQPGNSSLENLPPVAASIEQLLERQWSEGQQFLLEQGTPSDILGMLKSLHQLQVENRRLEEQIKNLTAKKERLQL
LNAQLSVPFPTITANPSPSHQIHTFSAQTAPTTDSLNSSKSPHIGNSFLPDNSLVPLNQDLTSSGQSTSSSSALSTPPPAGC
SPAQQGSGVSGVQQVNGVTVGALASGMQPVTSTIPAVSAVGGIIGALPGNQLAINGGGALNGVMQTPVTMSQNPT
PLTHTTVPPNATHPMPATLTNSASGLGLLSDQQRQILIHQQQFQQLLNSQQLTPVHRPHHFTQLPPTHFSPSM

B

Dot1l Histone H3 methyltransferase DOT1 variant c
Mass: 129542 Score: 67 Queries matched: 3 emPAI: 0.02

Query	Observed	Mr(expt)	Mr(calc)	ppm	Miss	Score	Expect	Rank	Peptide
1557	399.2108	1194.6107	1194.6105	0.18	0	34	0.0087	1	K.EISAHNQQLR.E
1795	492.6180	1474.8321	1474.8296	1.69	0	23	0.16	1	R.IVSSKPFAPLNFR.I
1795	568.2935	1134.5725	1134.5703	1.93	0	43	0.0025	1	R.NLSDIGTIMR.V

MGEKLELRLKSPVGAEPAVYPWPLPVYDKHHDAAHEIIETIRWVCEEIPDLKLAMENYVLIDYDTKSFESMQRLCDKY
RAIDSIHQLWKGTTQPMKLNTRPSNGLLRHILQQVYNHSVTDPEKLNNYEPFSPEVYGETSFDLVAQMIDEIKMTEDI
FVDLGSGVGQVVLQVAAATNCKHHYGVEKADIPAKYAETMDREFRKWMKWYGKKHAEYTLERGDFLSEEWRERI
TSVIFVNNFAFGPEVDHQLKERFANMKEGGRIVSSKPFAPLNFRINSRNLSDIGTIMRVVELSPLKGSVSWTGKPVSYYL
HTIDRTILENYFSSLKNPKLREEQEAARRRQQRENKSNATTPTKVPESKAAATEAPADSGAEEEKSGVATVKKPSPSKA
KKKLNKKGRKMAGRKRGRPKKMSAASAERKSKKSQSTLDLLHSPPPAPPSASPQDAYRAPHSPFYQLPPSTQLHSPN
LVAPTPPALQKLLESFRIQYLQFLAYTKTPQYKANLQQLLDQEKEKNTQLLGTAQQLFGHCQAQKEEIRRLFQQKLDELG
VKALTYNDLIQACKEISAHNQQLREQSEQLEKDNSELRSQSLRLLRARCEELRLDWSTLSLENLRKEKQALRSQISEKQR
CLELQISIVELEKTQRQQELLQLKSCVPPDDALSLHLRGKGALGRELEADAGRLRLELDCAKISLPHLSSMSPELSMNGH
ASYELCNAASRPSSKQNTPQYLASPLDQEVVPCTPSHSGRPRLEKLSGLALPVTRLSPAKIVLRRHLSQDHTGASKAATS
EPHPRPEHPKESSLPYQSPGLSNSMKLSPQDPPLASPATSPLTSEKGSEKGVKERAYSSHGETITSLPVSIPLSTVQPNKL
VSIPLASVVLPSRAERARSTPSPVPQPRDSSATLEKQTGASAHGAGGAGAGSRSLAVAPTGFYAGSVAISGALASSPA
ASGMESAVFDESSGPSSLFATMGSRSTPPQHPPLLSQSRNSGPASPAHQLTASPRLSVTTQGSLPTSKGELPSDPAFSD
PESEAKRRIVFSISVGASSKQSPSTRHSPLTSGTRGDCVQSHGQDSRKRSRRKRASAGTPSLSTGVSPKRRALPTVAGL
QSSGSPLNLNSMVSNINQPLEITAISSPESSLKSSPTPYQDHDQPPVLRKERPLGLTNGAHYSPLTSDEEPGSEDEPSSA
ERKIATISLESKSPPKTLENGGGLVGRKSAPSSEPINSSKWKSTFSPISDLGLAKAVDSPLQAGSALSHSPLFSFRPSLEPAA
EAKLPTHPRKSFAGSLGAAEGPSPGTNPPNGLAFSGGLAADLGLHSFNDGASLSHKGPDVTGLSASLSFPSQRGKDST
EANPFLSRRQPEGLGGLKGEGNANKESGESLPLCGPSDKASLPHGSRASKGRDRELDFKGGHNLFISAAAVPPGGLLG
PGLVTVASSAGSATPTAQAPRPFLSTFAPGPQFTLGPMSLQANLGSVAGSSVLQSLFSTVPAAAGLVHVSSTATRLTN
HTMGSFSSGVAGGTVGGN

Figure S1. Peptides identified and coverage of (A) Mllt10 and (B) Dot1l in Tcf4 complex in mouse small intestinal crypt. Amino acid sequences of Mllt10 and Dot1l detected in the Tcf4 immunoprecipitate from crypt lysates are underlined.
