# Supplementary material for: The Leukemia-Associated Mllt10/Af10-Dot1l Are Tcf4/β-Catenin Coactivators Essential for Intestinal Homeostasis
Source: PLoS Biol. 2010 Nov 16;8(11):e1000539. doi: 10.1371/journal.pbio.1000539 (PMC2982801; doi:10.1371/journal.pbio.1000539)
Supplement: Figure S2 — Specific enrichment of Mllt10 and Dot1l, and H3K79 di-/tri-methylation at c-Myc locus in mouse crypts. (A) Schematic representation of the mouse amplicons scanned in ChIP experiments by qPCR. Purified crypt and villus fractions from mouse intestine were subjected to ChIP using antibodies directed against Tcf4 (B), β-catenin (C), Mllt10/Af10 (D), Dot1l (E), di-methyl H3K79 (F), and tri-methyl H3K79 (G). Chromatin was immunoprecipitated with the specified antibodies followed by qPCR using primer pairs spanning the c-Myc locus as indicated in (A). Results are presented as percent immunoprecipitated over input and are representative of three independent experiments. (0.07 MB PDF) [file pbio.1000539.s002.pdf]

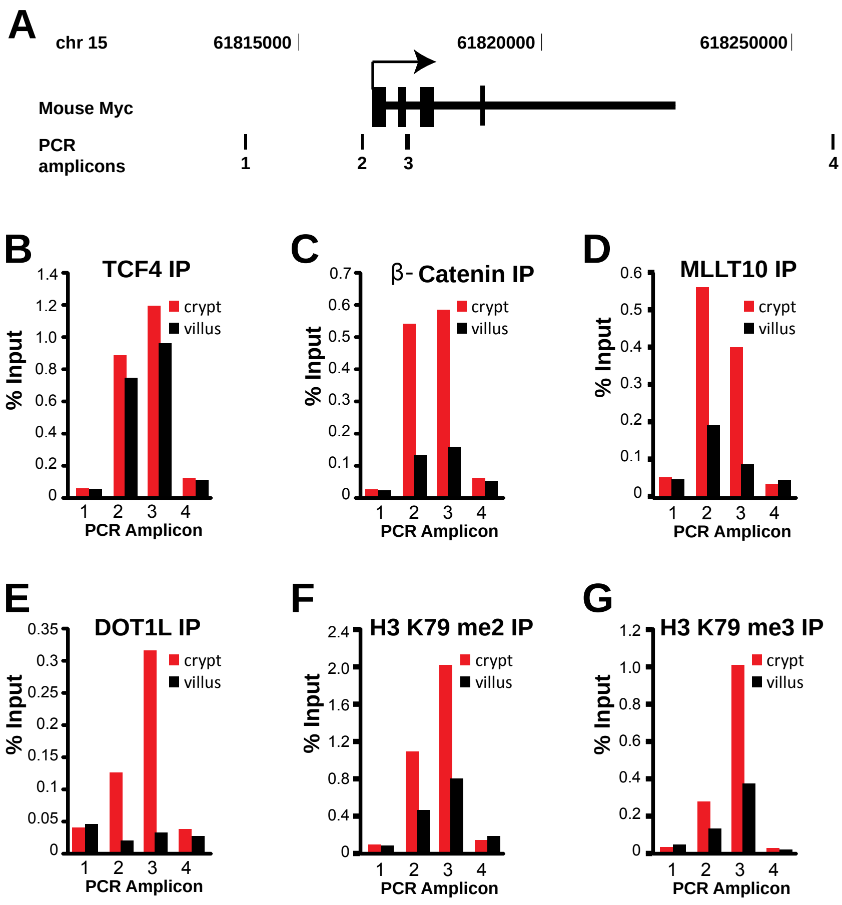

Figure S2. Specific enrichment of Mllt10 and Dot1l, and H3K79 di-/tri-methylation at *c-Myc* locus in mouse crypts. (A) Schematic representation of the mouse amplicons scanned in ChIP experiments by qPCR. Purified crypt and villus fractions from mouse intestine were subjected to ChIP using antibodies directed against Tcf4 (B),β-catenin (C), Mllt10/Af10 (D), Dot1l (E), dimethyl H3K79 (F) and trimethyl H3K79 (G). Chromatin was immunoprecipitated with the specified antibodies followed by qPCR using primer pairs spanning the *c-Myc* locus as indicated in (A). Results are presented as percent immuno-precipitated over input and are representative of three independent experiments.
