# Supplementary material for: The Leukemia-Associated Mllt10/Af10-Dot1l Are Tcf4/β-Catenin Coactivators Essential for Intestinal Homeostasis
Source: PLoS Biol. 2010 Nov 16;8(11):e1000539. doi: 10.1371/journal.pbio.1000539 (PMC2982801; doi:10.1371/journal.pbio.1000539)
Supplement: Figure S3 — β-catenin recruits Mllt10/Af10 to Wnt targets. (A) Ls174T CRC lysates were immunoprecipitated with antibodies against TCF4 (left panel) and β-catenin (right panel) and analyzed by Western blotting with the indicated antibodies for binding to MLLT10/Af10 and DOT1L. (B–H) β-catenin-dependent H3K79 methylation and recruitment of MLLT10 and DOT1L to c-MYC gene in Ls174T CRC. (B) Schematic representation of human c-MYC locus and amplicons scanned in ChIP assays by qPCR. ChIP experiments in Ls174T CRC uninduced or induced with Dox using antibodies against (C) TCF4, (D) β-catenin, (E) MLLT10, (F) DOT1L, (G) H3K79 dimethyl, and (H) H3K79 trimethyl. Immunoprecipitated DNA was analyzed by qPCR using primer pairs specific for the c-MYC locus as indicated. Results are presented as percent immunoprecipitated over input and are representative of three independent experiments. (0.08 MB PDF) [file pbio.1000539.s003.pdf]

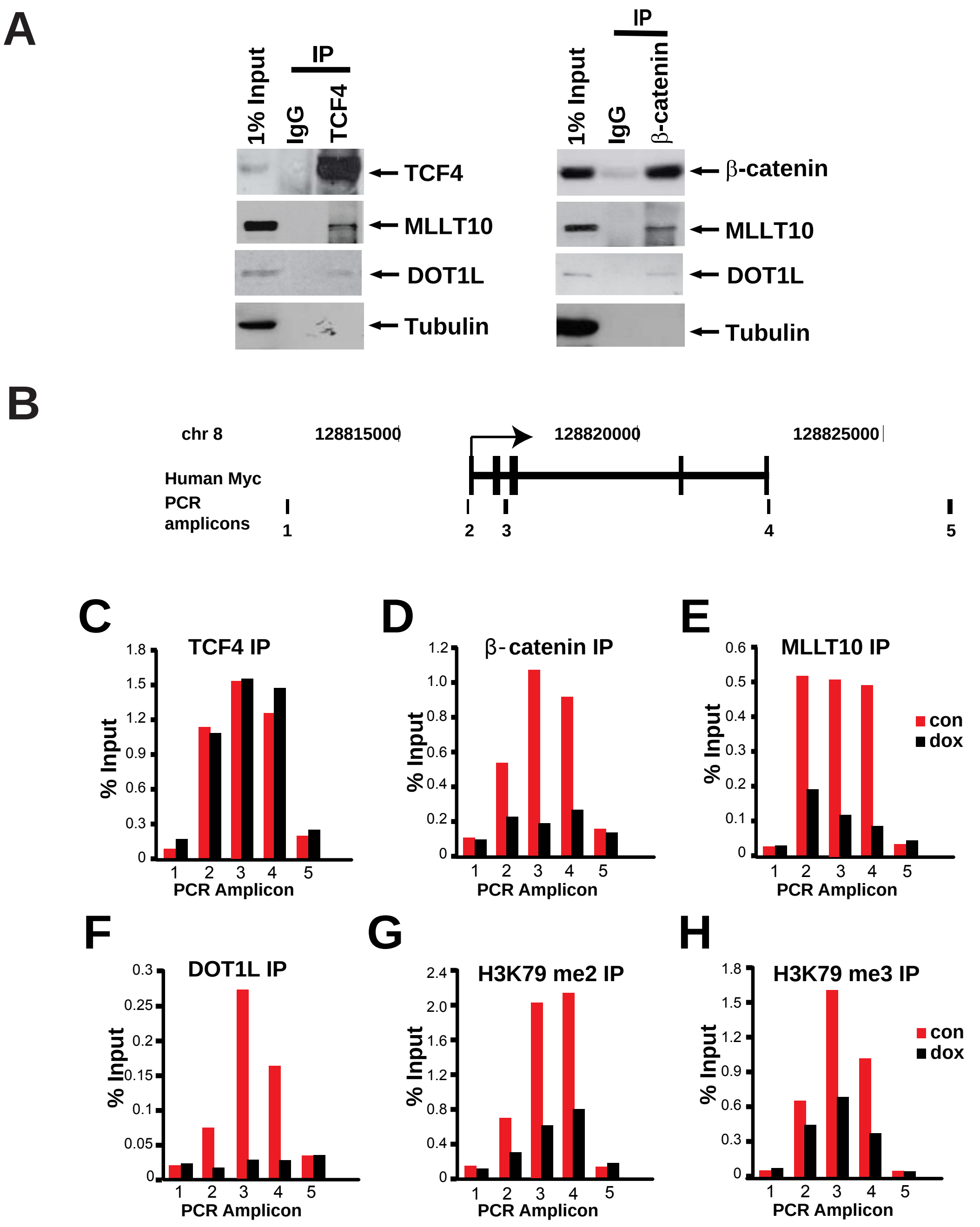

Figure S3. β-catenin directly recruits Mllt10/Af10 to Wnt targets. (A) Ls174T CRC lysates were immunoprecipitated with antibodies against TCF4 (left panel) and β-catenin (right panel) and analyzed by Western blotting with the indicated antibodies for binding to MLLT10/Af10 and DOT1L. (B-H) β-catenin-dependent H3K79 methylation and recruitment of MLLT10 and DOT1L to *c-MYC* gene in Ls174T CRC. B) Schematic representation of human *c-MYC* locus and amplicons scanned in ChIP assays by qPCR. ChIP assays in Ls174T CRC uninduced or induced with Dox using antibodies against (C) TCF4 (D) β-catenin (E) MLLT10 (F) DOT1L (G) H3K79 dimethyl and (H) H3K79 trimethyl. Immunoprecipitated DNA was analyzed by qPCR using primer pairs specific for the *c-MYC* locus as indicated. Results are presented as percent immunoprecipitated over input and are representative of three independent experiments.
