# Supplementary material for: The Leukemia-Associated Mllt10/Af10-Dot1l Are Tcf4/β-Catenin Coactivators Essential for Intestinal Homeostasis
Source: PLoS Biol. 2010 Nov 16;8(11):e1000539. doi: 10.1371/journal.pbio.1000539 (PMC2982801; doi:10.1371/journal.pbio.1000539)
Supplement: Figure S4 — Mllt10/Af10 interacts directly with β-catenin. (A) Recombinant GST-fused TCF4 and β-catenin proteins were used in pull-down assays with in vitro translated S35 labeled MLLT10 to examine direct interaction. (B) Schematic representation of N-Terminal and C-Terminal MLLT10 deletion mutants and S35 labeled ΔC- and C-Terminal β-catenin deletion mutants used in GST pulldown assays (C). N-Terminal MLLT10 interacts directly with the β-catenin C-terminal domain. (0.03 MB PDF) [file pbio.1000539.s004.pdf]

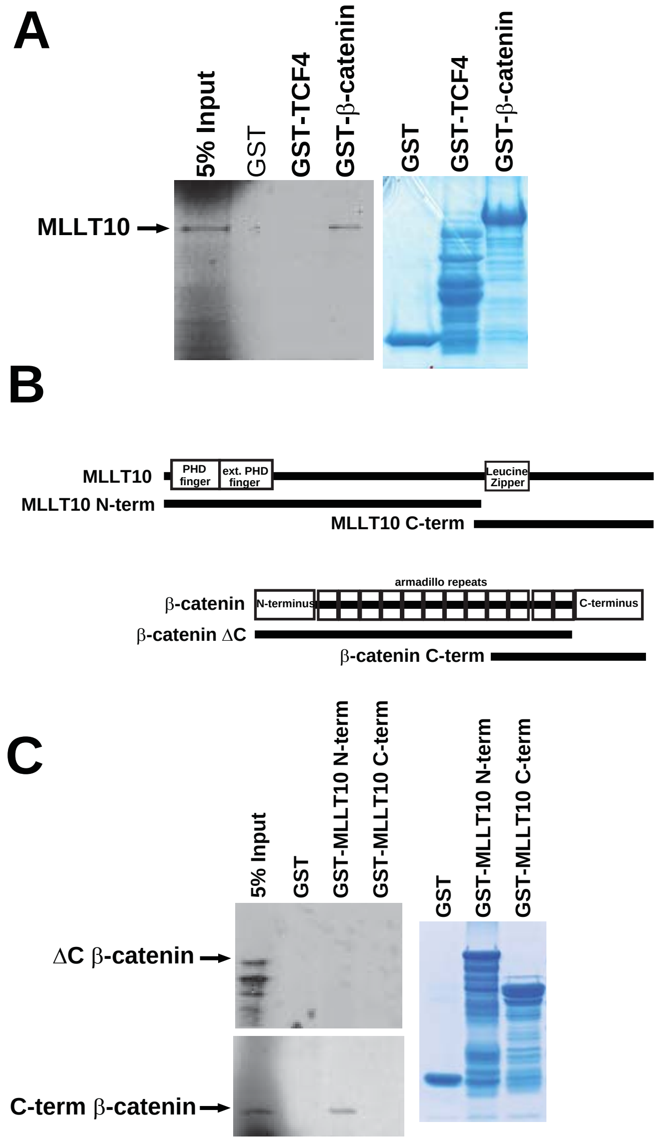

Figure S4. Mllt10/Af10 interacts directly with β-catenin. (A) Recombinant GST-fused TCF4 and β-catenin proteins were used in pull-down assays with in vitro translated S35 labeled MLLT10 to examine direct interaction. (B) Schematic representation of N-Terminal and C-Terminal MLLT10 deletion mutants and S35 labeled ΔC- and C-Terminal β-catenin deletion mutants used in GST pulldown assays (C). N-Terminal MLLT10 interacts directly with the β-catenin C-terminal domain.
