# Supplementary material for: The Leukemia-Associated Mllt10/Af10-Dot1l Are Tcf4/β-Catenin Coactivators Essential for Intestinal Homeostasis
Source: PLoS Biol. 2010 Nov 16;8(11):e1000539. doi: 10.1371/journal.pbio.1000539 (PMC2982801; doi:10.1371/journal.pbio.1000539)
Supplement: Figure S5 — (A) Significant overlap between differentially regulated genes in response to MLLT10 or DOT1L depletion in HEK293T cells after Wnt stimulation. Comparison of the corresponding expression pattern after siRNA suppression of MLLT10/AF10 or DOT1L in Wnt induced condition. Heatmap showing 1,116 transcripts after siRNA depletion of MLLT10 and 9 h Wnt stimulation in HEK293T cells with greater than 1.5-fold variation. Also shown is comparison of the corresponding expression pattern of genes induced after 9 h Wnt treatment and after siRNA suppression of DOT1L. Red, upregulated after MLLT10 suppression; green, downregulated after MLLT10 suppression; grey, missing data. (B) Venn diagram comparatively depicting genes suppressed upon MLLT10 and DOT1L depletion in HEK293T cells under Wnt-induced conditions. (0.03 MB PDF) [file pbio.1000539.s005.pdf]

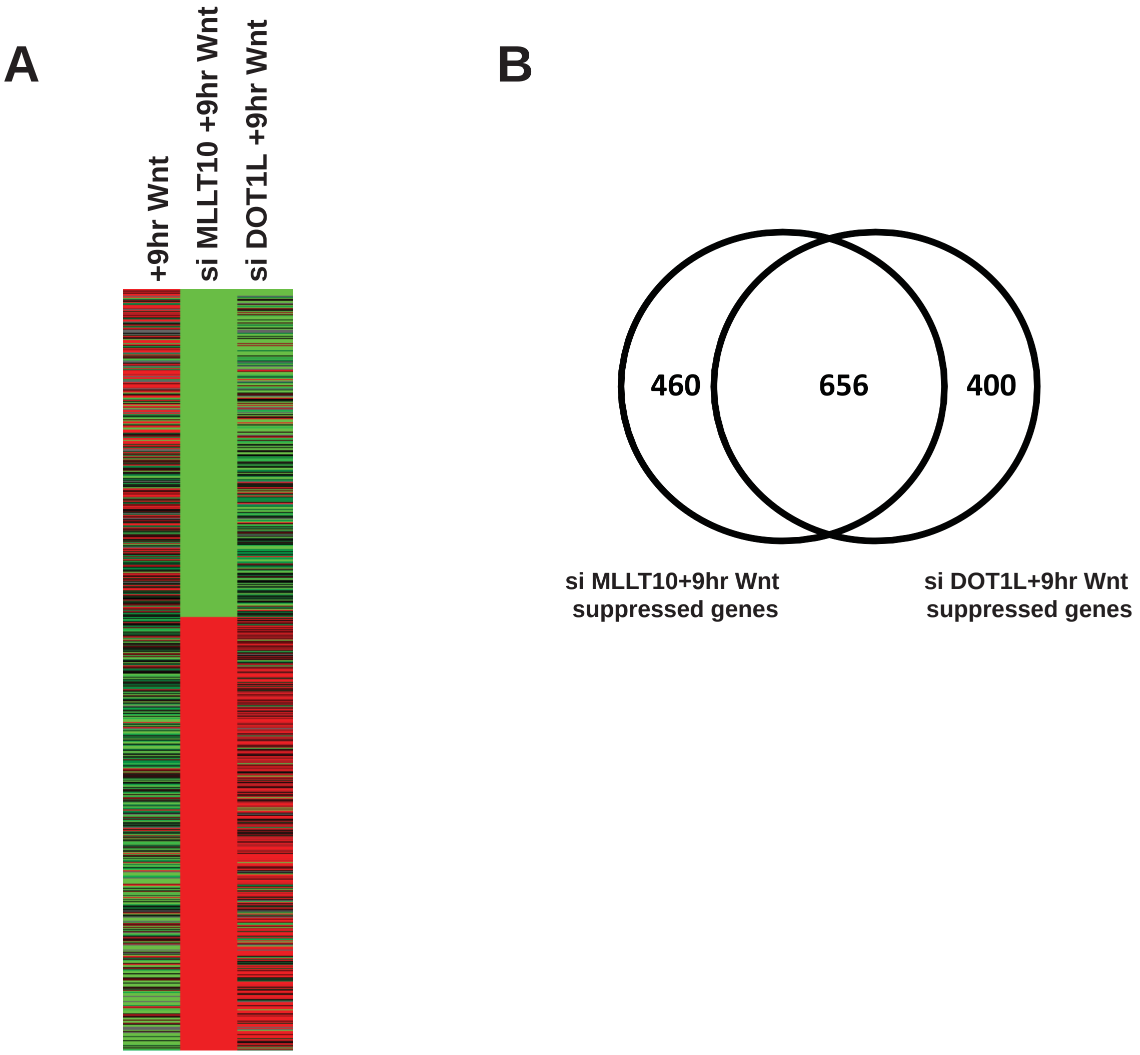


**Figure S5. (A) Large overlap between differentially regulated genes in response to MLLT10 or DOT1L depletion in HEK293T cells after Wnt stimulation.**
