# Supplementary material for: The Leukemia-Associated Mllt10/Af10-Dot1l Are Tcf4/β-Catenin Coactivators Essential for Intestinal Homeostasis
Source: PLoS Biol. 2010 Nov 16;8(11):e1000539. doi: 10.1371/journal.pbio.1000539 (PMC2982801; doi:10.1371/journal.pbio.1000539)

Figure S7. Depletion of *mllt10* and *dot1l* rescues *axin2* mis-expression in *apc mcr/mcr* zebrafish, mimicking *tcf7l2* depletion.

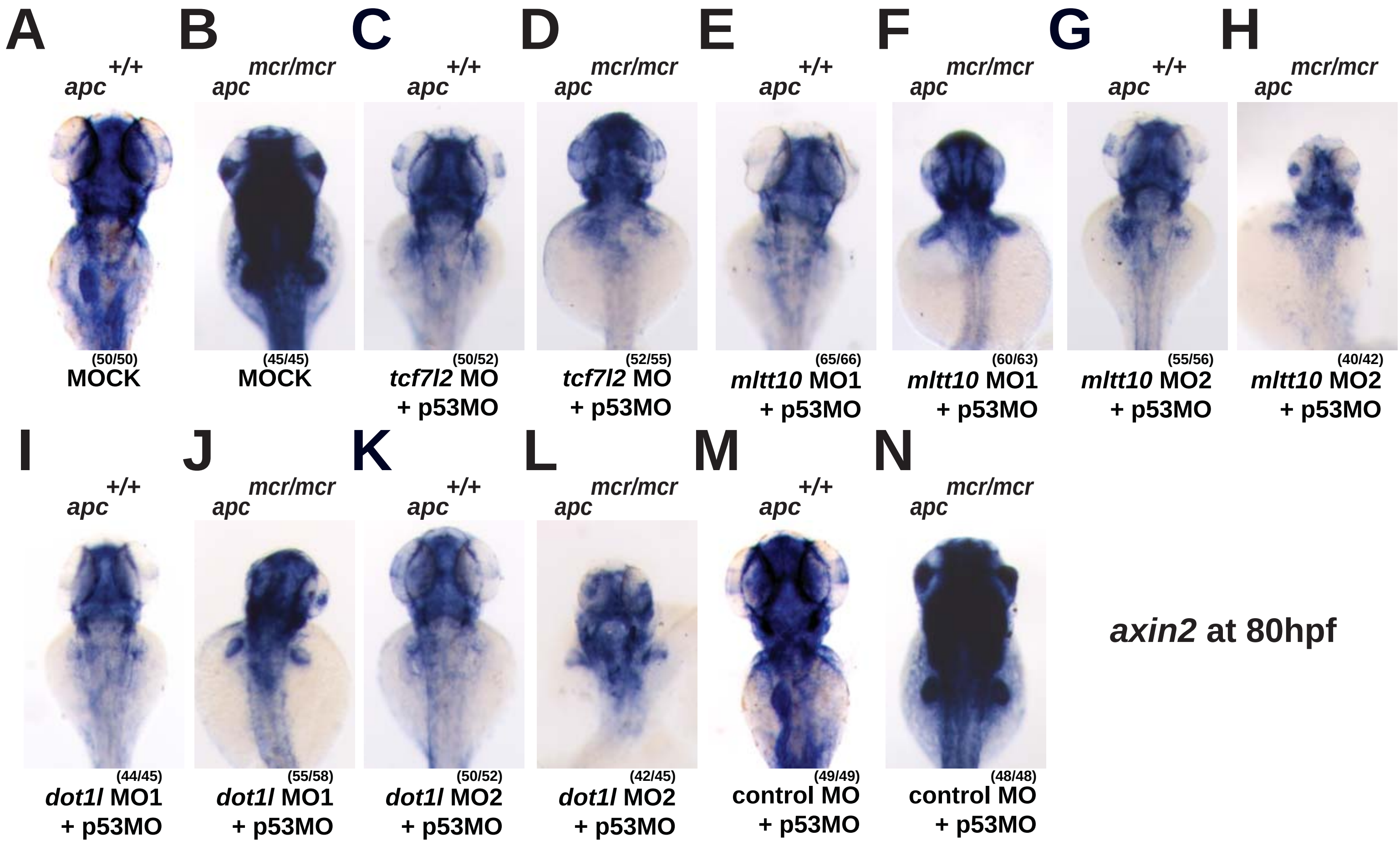

Supplement: Figure S7 — Depletion of tcf7l2, mllt10/af10 and dot1l rescues mis-expression of axin2 in apcmcr/mcr zebrafish, placing these genes downstream of Apc as Wnt target gene activators (A–N). Representative whole mount in situ hybridizations for axin2 in wild type and apcmcr/mcr mutant embryos at 80 hpf injected with (A,B) buffer alone, (C,D) MO against tcf7l2, (E–H) two independent mllt10/af10 MOs, (I–L) two independent dot1l MO, and (M,N) control MO. All MOs have been coinjected with a MO against p53. All images were captured using the same exposure and represent at least three independent experiments. In parentheses number of embryos showing described phenotype per number of total embryos analyzed. (0.06 MB PDF) [file pbio.1000539.s007.pdf]
