# Supplementary material for: The Leukemia-Associated Mllt10/Af10-Dot1l Are Tcf4/β-Catenin Coactivators Essential for Intestinal Homeostasis
Source: PLoS Biol. 2010 Nov 16;8(11):e1000539. doi: 10.1371/journal.pbio.1000539 (PMC2982801; doi:10.1371/journal.pbio.1000539)

Figure S8. Depletion of *tcf7l2*, *mllt10* or *dot1l* does not affect expression levels of other *tcf/lef* members in zebrafish embryos.

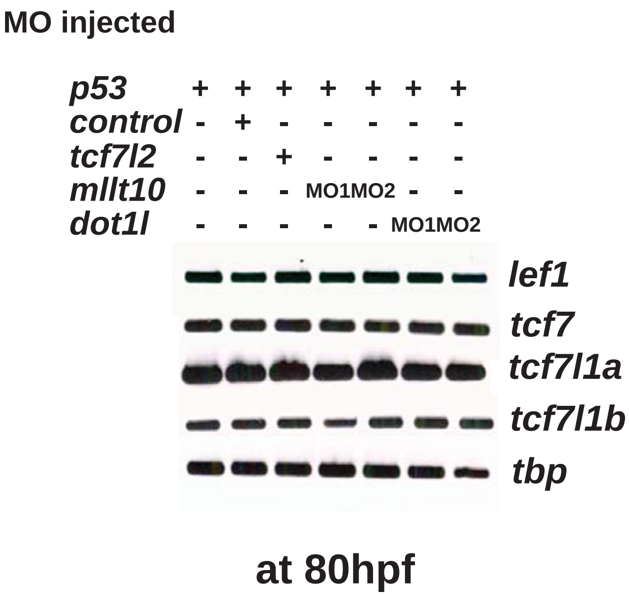

Supplement: Figure S8 — Depletion of tcf7l2 , mllt10 , or dot1l does not affect the expression levels of other tcf/lef family members in zebrafish embryos. RT-PCR analysis of lef1, tcf7, tcf7l1a, tcf7l1b, and tbp RNA expression levels in whole embryos injected with MO against p53 alone, or MO against p53 coinjected with MOs against tcf7l2, mllt10, dot1l, or control MO, respectively. (0.02 MB PDF) [file pbio.1000539.s008.pdf]
