# Supplementary material for: The Leukemia-Associated Mllt10/Af10-Dot1l Are Tcf4/β-Catenin Coactivators Essential for Intestinal Homeostasis
Source: PLoS Biol. 2010 Nov 16;8(11):e1000539. doi: 10.1371/journal.pbio.1000539 (PMC2982801; doi:10.1371/journal.pbio.1000539)
Supplement: Figure S6 — mRNA expression of tcf7l2 , mllt10 , and dot1l during zebrafish embryonic development and their depletion by splice-inhibiting MO sequences. (A) Phylogenetic tree analysis places zebrafish (Danio rerio) Mllt10 close to mouse and human sequences. (B–C) Amino acid sequence alignment of the Leucine zipper and PHD finger domains show high conservation of Mllt10 between species. (D) RT-PCR analysis of tcf7l2, mllt10, dot1l, and tbp RNA expression levels in whole embryos at different stages of embryonic development. (E) Representation of the splice-blocking MO sequences (blue) generated to deplete mRNA levels for tcf7l2, mllt10, and dot1l. (0.03 MB PDF) [file pbio.1000539.s006.pdf]

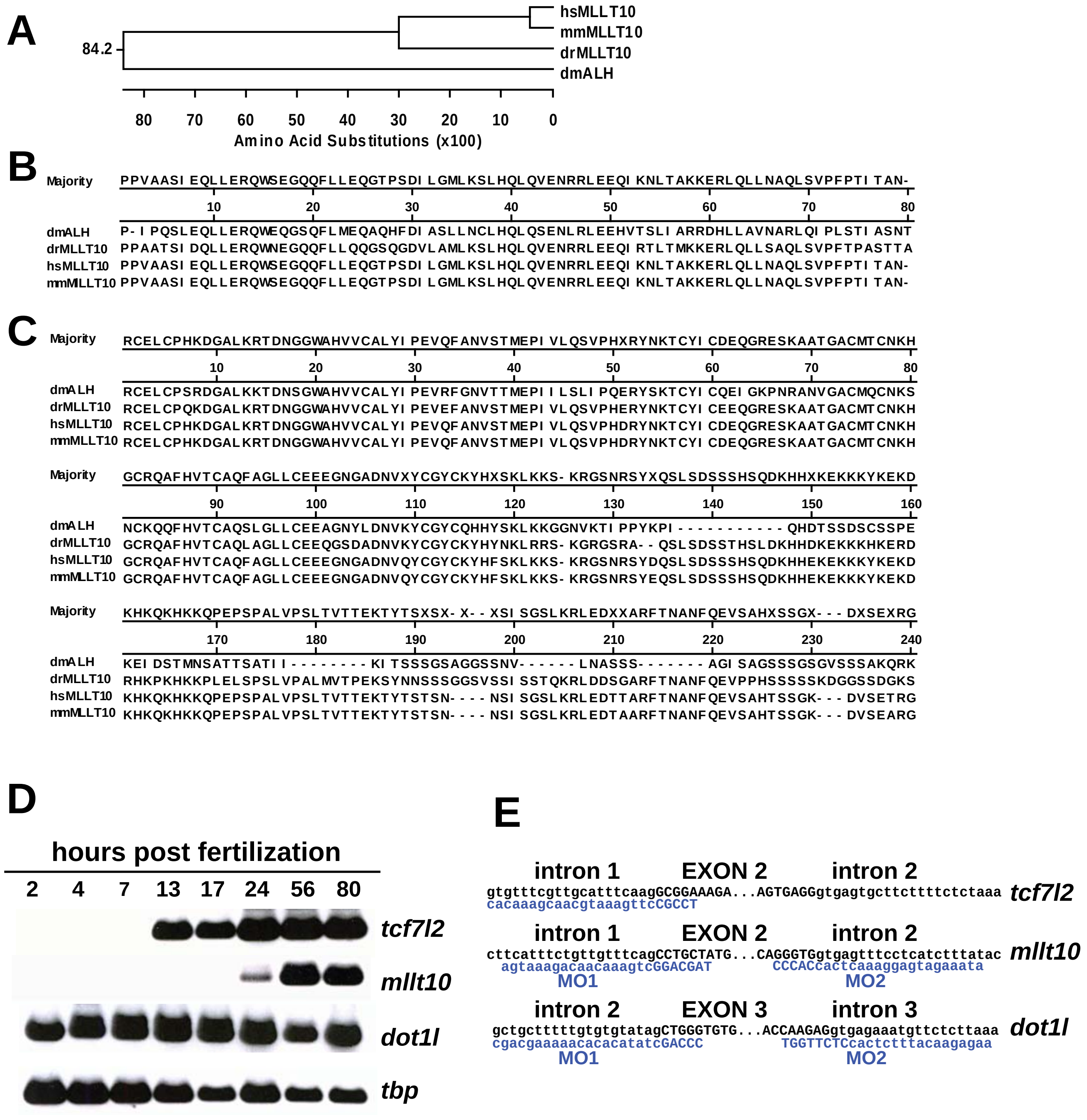

Figure S6. mRNA expression of *tcf7l2, mllt10 and dot1l* during zebrafish embryonic development and their depletion by splice-inhibiting MO sequences.
